# Genome wide distribution of G-quadruplexes and their impact on gene expression in malaria parasites

**DOI:** 10.1371/journal.pgen.1008917

**Published:** 2020-07-06

**Authors:** Elodie Gazanion, Laurent Lacroix, Patrizia Alberti, Pratima Gurung, Sharon Wein, Mingpan Cheng, Jean-Louis Mergny, Ana Rita Gomes, Jose-Juan Lopez-Rubio

**Affiliations:** 1 MIVEGEC UMR IRD 224, CNRS 5290, Montpellier University, Montpellier, France; 2 IBENS, Ecole Normale Supérieure, CNRS, Inserm, PSL Research University, Paris, France; 3 "Structure and Instability of Genomes" laboratory, Muséum National d'Histoire Naturelle (MNHN), Inserm U1154, CNRS UMR 7196, Paris, France; 4 Laboratory of Pathogen-Host Interactions (LPHI), UMR5235, CNRS, Montpellier University, Montpellier, France; 5 ARNA Laboratory, IECB, CNRS UMR5320, INSERM U1212, Bordeaux University, Pessac, France; 6 Institute of Biophysics of the Czech Academy of Sciences, Czech Republic; 7 Laboratoire d’Optique et Biosciences, Ecole Polytechnique, CNRS, INSERM, Institut Polytechnique de Paris, France; Imperial College London, UNITED KINGDOM

## Abstract

Mechanisms of transcriptional control in malaria parasites are still not fully understood. The positioning patterns of G-quadruplex (G4) DNA motifs in the parasite’s AT-rich genome, especially within the *var* gene family which encodes virulence factors, and in the vicinity of recombination hotspots, points towards a possible regulatory role of G4 in gene expression and genome stability. Here, we carried out the most comprehensive genome-wide survey, to date, of G4s in the *Plasmodium falciparum* genome using G4Hunter, which identifies G4 forming sequences (G4FS) considering their G-richness and G-skewness. We show an enrichment of G4FS in nucleosome-depleted regions and in the first exon of *var* genes, a pattern that is conserved within the closely related *Laverania Plasmodium* parasites. Under G4-stabilizing conditions, *i*.*e*., following treatment with pyridostatin (a high affinity G4 ligand), we show that a *bona fide* G4 found in the non-coding strand of *var* promoters modulates reporter gene expression. Furthermore, transcriptional profiling of pyridostatin-treated parasites, shows large scale perturbations, with deregulation affecting for instance the ApiAP2 family of transcription factors and genes involved in ribosome biogenesis. Overall, our study highlights G4s as important DNA secondary structures with a role in *Plasmodium* gene expression regulation, sub-telomeric recombination and *var* gene biology.

## Author summary

Malaria persists as a global health concern and is caused by divergent eukaryotic parasites of the genus *Plasmodium*. These parasites have a complex life cycle during which stage transitions are characterised by tightly regulated cascades that ensure stage-specific gene expression, which are not yet fully understood. Here, we have performed a genome wide scan of G-quadruplex (G4) DNA motifs in the parasite’s AT-rich genome, using G4Hunter. We highlight a significant enrichment of G4s in nucleosome-depleted regions and in the first exon of *var* genes, which encode an important virulence factor associated with severe malaria. This is a pattern conserved among *Plasmodium*-related species of the *Laverania* subgenus. We then validate the regulatory function of G4s on transcription, using a luciferase reporter system. Finally, we show that the highly selective G4-stabilizing ligand pyridostatin causes large scale perturbations of the transcriptome in *Plasmodium falciparum*, thus showcasing their potential as antimalarials. Altogether, our study highlights G4s as a new layer of gene regulation in malaria parasites, and a likely potentiator of *var* gene diversity.

## Introduction

Malaria parasites are responsible for half a million deaths worldwide each year, most of which are children [1]. The most severe form of malaria in humans is caused by *Plasmodium falciparum* parasites, which display a complex life cycle alternating between two different hosts, the female *Anopheles* mosquito and the human host. Life cycle progression is supported by timely regulation of gene expression, where the majority of the ~5,600 genes are developmentally regulated throughout the different stages [2]. During the pathogenic phase of the disease, parasites undergo a 48-hour cycle of vegetative growth within the human red blood cells (RBCs), during which parasites progress through ring, trophozoite and schizont stages to produce 16–32 merozoites, which are able to, in turn, invade new RBCs. How these developmental transitions are regulated at the molecular level is not yet fully elucidated. In these parasites, timely protein expression results from several layers of regulatory mechanisms, which include transcription activation by transcription factors, dynamics of RNA degradation, changes in nuclear organization, chromatin modifications, among others [3]. An example of this complex regulation is the mono-allelic expression of *var* genes that encode the *P*. *falciparum* Erythrocyte Membrane Protein 1 (PfEMP1) [4], which ensures host immunity evasion. The *var* genes superfamily of *P*. *falciparum* is composed of ~60 members, which share a conserved protein architecture and have a high level of sequence similarity in exon 2, while being variable in the first exon [5]. Specific epigenetic marks [6–8] and *var*-specific antisense transcription [9] ensure that only one *var* gene is expressed at a time [4]. The expression of these proteins on the RBC surface induces cytoadherence to endothelial cells and rosetting of uninfected erythrocytes, both associated with the high-rate mortality of the disease [10].

G-quadruplexes (G4s) are non-canonical structures that form in guanine-rich nucleic acids. These structures are stabilized by the stacking of G-quartets, planar arrays of four guanines that are held together by Hoogsteen base pairing in the presence of monovalent cations. G4s can adopt intramolecular and intermolecular structures formed by one or two or more DNA or RNA strands, respectively, in parallel, hybrid or antiparallel configurations [11]. Quadruplexes have been ascribed roles in fundamental biological processes, including gene expression regulation, replication, RNA processing, DNA recombination and telomere maintenance [12,13]. In the human genome, G4s are mainly associated with telomeric repeats and regulatory regions, such as transcription start sites (TSS), promoters, replication origins and nucleosome-depleted regions [11,14]. The characterization of intracellular factors that recognize and process these secondary structures together with the development of G4-stabilizing ligands able to stabilise G4 structures *in vivo*, led to a better understanding of their biological functions in the cell [15,16]. In human cells, the presence of G4 structures at telomeres and in the promoter of cancer-related genes, such as *c-MYC*, *hTERT*, *c-KIT* or *BCL2* has been shown to be involved in telomere regulation and in the transcriptional and/or translational control of these oncogenes [17–20]. Hence, G4s have attracted great attention as new anticancer therapeutic targets [20,21] and several G4 ligands showed antitumor effects, such as Quarfloxin which completed Phase II trials [22].

Occurrence of G4s in other organisms including yeast [23], bacteria [24,25], plants [26], viruses [27] and parasites [28,29] has been studied by bioinformatics surveys and physical characterization, mainly based on spectroscopic methods. Recently, the development of the G4-seq method that takes advantage of the ability of G-quadruplex structures to induce polymerase stalling allowed to identify G4 location using next-generation sequencing [30]. G4-seq unveiled a high proportion of non-canonical G-quadruplex structures that bear long loops or bulges, in several eukaryotic and prokaryotic genomes [31]. One leading question concerns the role of G4s in gene expression regulation in these organisms and their potential as novel therapeutic targets for those of medical importance. In *Neisseria gonorrhoeae*, the causative agent of Gonorrhea, G4s promote pilin antigenic variation through a RecA-mediated strand exchange [32]. In several viruses including HIV, Herpes viruses and Epstein-Barr, viral G4s present in promoters have been shown to modulate promoter activity [33–35]. Interestingly, several G4-stabilizing molecules that were initially developed as potential anticancer agents displayed antimalarial activity, although their precise molecular mode of action remains unknown [28,36–38].

In *P*. *falciparum*, because of the GC-poorness of its 23-Mb genome (80.6% A+T) [39], most of putative G4 forming sequences (G4FS) predicted by QGRS Mapper were found at telomeres which contain multiple repeats of the degenerate GGGTTYA motif (828 G4FS) [29], as compared to 80 G4FS found in non-telomeric regions [40]. This widely used algorithm maps a consensus G4 motif (four G-triplets linked by short nucleotide loops), but fails to identify non-canonical G4s, thus leading to missed G4s [41]. Interestingly, almost half of non-telomeric G4FS were found associated with *var* genes [29,40] and hypothesised to both promote DNA recombination on those sites [40,42] and affect gene expression of this major virulence factor [36,42,43].

Here, we present an extended analysis of the G4 distribution in *Plasmodium* using G4Hunter, an algorithm that allows an accurate prediction of sequences with G4-forming potential considering their G-richness and G-skewness, rather than a conservative consensus sequence [41]. Our results highlight a significant enrichment of G4 DNA motifs in *var* genes and in nucleosome-depleted regions of the *P*. *falciparum* genome. Importantly, the *var*-specific enrichment is conserved among *Plasmodium*-related species of the *Laverania* subgenus. Furthermore, we have characterized several G4FS by biophysical methods and functional assays and show that the highly selective G4 molecule pyridostatin [16] causes genome wide gene deregulation.

## Results

### Distribution of G4 forming sequences in the *P*. *falciparum* genome

Unlike G4-search algorithms that map a consensus G4 DNA motif such as Quadparser [44] or QGRS Mapper [45], G4Hunter computes a score for a given sequence taking into account its G-richness and G-skewness [41]. We applied it to the reference strain *P*. *falciparum* 3D7 (version 28 on PlasmoDB [46]) and obtained a total number of G4 forming sequences (G4FS) ranging from 1,763 to 145 (Table 1 and S1 Table), when applying a threshold of the score from 1.2 to 1.75 (a higher threshold corresponding to a more stringent search). We generated 3 lists of G4FS setting the threshold at 1.2, 1.5 and 1.75 that we named G4H1.2, G4H1.5 and G4H1.75 respectively. G4Hunter therefore unveils 2 to 22 more sequences than previously described in *P*. *falciparum* [40]. This corresponds to densities (G4FS/kb) of 0.076 for G4H1.2 and 0.019 for G4H1.5 (Table 1), which are lower (by 1.6-fold) than in *Dictyostelium discoideum*, an organism whose genome harbours a similar high AT content (78%) [47,48].

**Table 1:** Number and density of G4FS that are present in different genome features in *P*. *falciparum* and in related species of *Plasmodium* predicted with G4Hunter. For each region, the total number of G4 motifs overlapping to the region intervals was calculated.

G4H threshold		G4H1.2		G4H1.5		G4H1.75	
		n	Density^a^	n	Density^a^	n	Density^a^
*P*. *falciparum*	THSS^b^	341	0.695	109	0.213	43	0.084
	Promoter^c^	288	0.032	88	0.011	41	0.005
	Transcript^d^	1,148	0.082	260	0.019	86	0.006
	Exon	1,111	0.089	250	0.020	83	0.007
	Intron	68	0.049	16	0.012	3	0.002
	Intergenic	642	0.069	187	0.020	62	0.007
	TSS^e^	61	0.117	12	0.023	4	0.008
	*var* genes^f^	376	0.782	126	0.262	54	0.112
	**Whole genome**^**g**^	**1,763**	**0.076**	**441**	**0.019**	**145**	**0.006**
*P*. *adleri*	Promoter	231	0.027	85	0.009	30	0.003
	Transcript	611	0.044	120	0.009	30	0.002
	Exon	583	0.047	108	0.009	28	0.002
	Intron	51	0.040	13	0.010	3	0.002
	Intergenic	299	0.035	111	0.013	38	0.005
	*var* genes	125	0.358	33	0.095	12	0.034
	**Whole genome**	**894**	**0.044**	**228**	**0.011**	**67**	**0.003**
*P*. *billcollinsi*	Promoter	219	0.025	73	0.008	35	0.004
	Transcript	942	0.071	195	0.015	67	0.005
	Exon	877	0.074	177	0.015	60	0.005
	Intron	100	0.070	25	0.017	10	0.007
	Intergenic	320	0.033	112	0.011	51	0.005
	*var* genes	251	1.073	87	0.372	40	0.171
	**Whole genome**	**1,248**	**0.057**	**305**	**0.014**	**118**	**0.005**
*P*. *blacklocki*	Promoter	228	0.028	76	0.010	34	0.004
	Transcript	556	0.042	82	0.006	17	0.001
	Exon	519	0.044	71	0.006	11	0.001
	Intron	64	0.046	14	0.010	7	0.005
	Intergenic	300	0.034	98	0.011	39	0.004
	*var* genes	73	0.494	11	0.074	3	0.020
	**Whole genome**	**843**	**0.041**	**178**	**0.009**	**56**	**0.003**
*P*. *reichenowi*	Promoter	256	0.029	91	0.011	38	0.004
	Transcript	1,433	0.098	393	0.027	139	0.010
	Exon	1,369	0.105	372	0.029	128	0.010
	Intron	96	0.061	26	0.016	13	0.008
	Intergenic	367	0.038	131	0.013	49	0.005
	*var* genes	607	0.778	242	0.310	103	0.132
	**Whole genome**	**1,773**	**0.077**	**520**	**0.023**	**188**	**0.008**
*P*. *praefalciparum*	Promoter	255	0.027	90	0.010	38	0.004
	Transcript	1,046	0.071	205	0.014	58	0.004
	Exon	1,005	0.076	195	0.015	56	0.004
	Intron	75	0.050	18	0.012	3	0.002
	Intergenic	357	0.035	124	0.012	50	0.005
	*var* genes	227	0.473	63	0.131	24	0.050
	**Whole genome**	**1,383**	**0.060**	**321**	**0.014**	**107**	**0.005**

^a^ Density represents the number of G4FS per kilobase (kb). Density was calculated by dividing the number of G4FS by the total feature length and multiplying by 1,000.

^b^ Tn5 transposase hypersensitivity sites (THSS) correspond to chromatin accessibility (data from ATAC-Seq experiments). Genomic coordinates were obtained from Ruiz *et al*. [49].

^c^ Promoter regions were arbitrary defined as 2 kb upstream of the start codon.

^d^ The number of G4FS in the feature “transcript” may not be equal to exon plus intron regions when G4FS were found at the exon/intron junction, in which case they were counted as being in both “exon” and “intron” features.

^e^ We used transcription start sites (TSS) coordinates from Adjalley *et al*. [50].

^f^ Transcript coordinates of 60 *var* genes.

^g^ Number of G4FS represent the total number of G4 sequences present in the genome.

**Fig 1:**
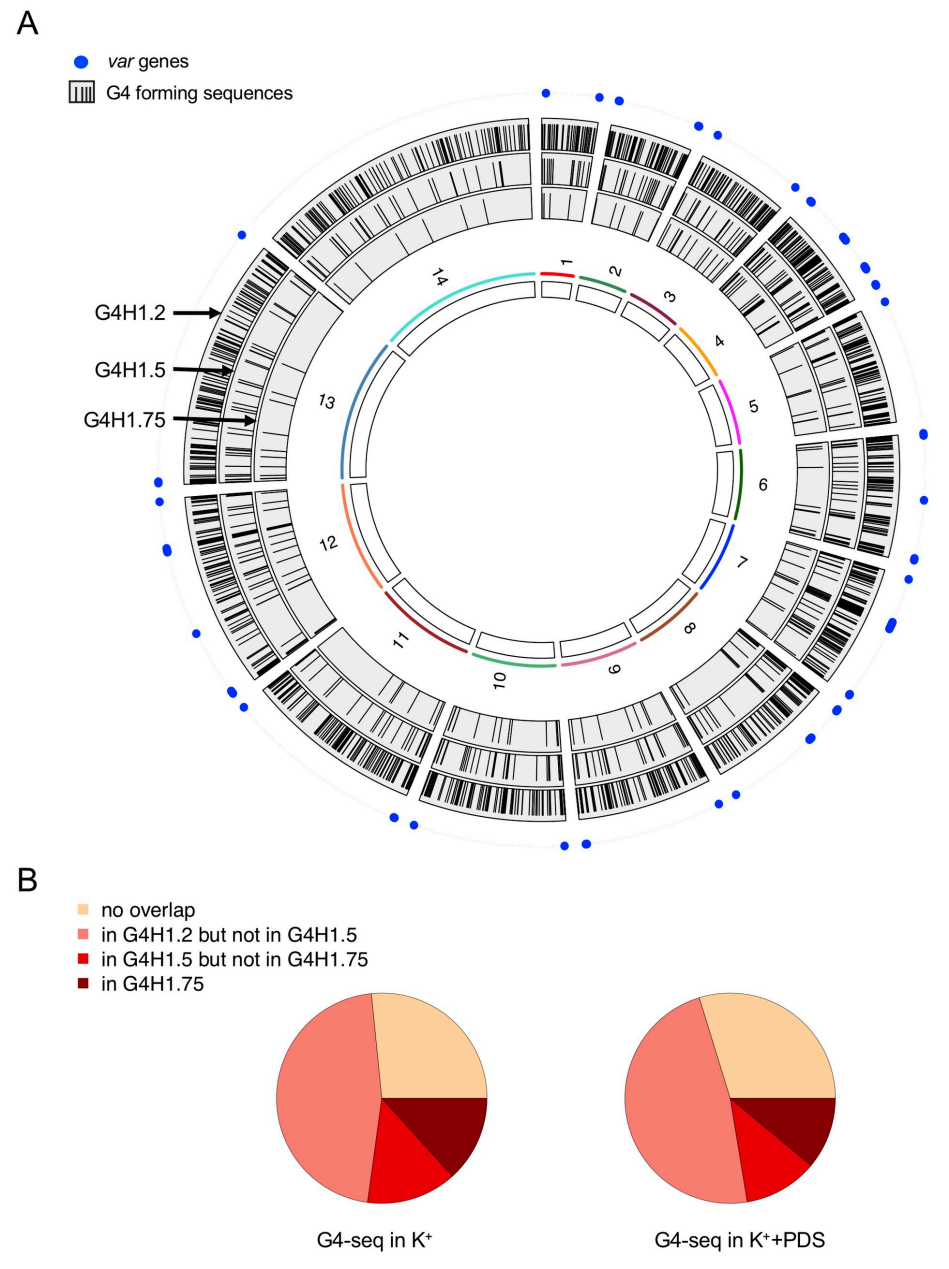
Distribution of G4FS across the *P*. *falciparum* 3D7 nuclear genome. (A) The positions of G4FS using G4Hunter at threshold 1.2 (outer track), 1.5 (middle track) and 1.75 (inner track) are represented as black bars. The 14 chromosomes are represented in white rectangles on the inner track. Blue filled circles represent *var* genes location. (B) Number of overlapping G4FS sequences between G4Hunter and G4-seq in K^+^ (left panel) and K^+^+PDS conditions (right panel) [31], according to the G4Hunter score.

G4FS were found on all chromosomes, with some chromosomic regions displaying higher G4 density, for instance chromosome ends and central regions (Fig 1A). Indeed, chromosome ends in *Plasmodium* are capped by telomeric repeats prone to G4 formation [51,52].

In agreement with previous studies [29,40], G4FS with score of 1.2 or higher co-localize with the multigene *var* family that are found in subtelomeric regions and in internal gene clusters on chromosomes 4, 7, 8 and 12 (Fig 1A). Similar distribution patterns were observed with scores of 1.5 and 1.75 (Fig 1A), indicating that sequences with higher propensity to form G4s are mainly found in these regions. Furthermore, G4FS were slightly enriched on chromosome 4 (S1 Fig) where a higher number of *var* genes is present on the minus strand (S2A Fig), compared to global gene distribution, where no significant strand occupancy or chromosome bias was observed (S2B Fig).

We also compared our G4 dataset with the recently published list of G4 structures identified *in vitro*, in the *P*. *falciparum* genome using the G4-seq method [31]. This method relies on the detection of base calling errors triggered by G4-mediated DNA polymerase stalling during sequencing reactions, under G4-promoting conditions (*i*.*e*. in the presence of K^+^ with or without the stabilizing ligand pyridostatin (PDS)) [30]. In *P*. *falciparum*, a total of 173 and 326 observed G-quadruplexes (OQs) were identified in presence of K^+^ and K^+^+PDS, respectively [31]. Among those, we found that 73% of OQs in K^+^ (127/173) and 70% of OQs in K^+^+PDS (229/326) were predicted by G4Hunter at a threshold of 1.2 (minimum overlap = 1 nt). At our most permissive threshold (G4H1.2) half of the OQs in both conditions overlapped only with G4H1.2 (Fig 1B). On the other hand, only 25% of our putative G4FS at threshold 1.75 were confirmed by G4-seq (S2 Table). To better understand this discrepancy between G4FS and OQs, we further examined the OQs that were missed by G4Hunter. Among those, 33% (32/97) of OQs exhibited a G4Hunter score below 0.5 and therefore highly unlikely to form G4 structures (Table 2). In fact, sequence analysis revealed that some OQs were highly AT-rich with long stretches of A or T, while others contained GA or GAA motifs (Table 2). As it is rather difficult to imagine G4 formation with a DNA sequence containing a few G or no G at all, we consider therefore these candidates as false-positive G4s.

**Table 2:** Observed G-quadruplexes (OQs) identified by G4-seq [31] that do not overlap with G4H1.2 and that exhibited a G4Hunter score below 0.5. DNA sequences and the score of the “best” G4 identified by G4Hunter in these G4-seq hits are shown. OQs with GA/GAA motifs or with AT repeats are highlighted in bold and in grey, respectively.

Chromosome	G4 sequence^a^	Strand	Length	G4Hunter score
Pf3D7_01_v3	TTTTTTTTTTTTTACTTCTTTCGTC	+	25	0.12
Pf3D7_01_v3	TAAATAATAAAGACAATATATTAAACATAT	-	45	0.08
Pf3D7_01_v3	**GAAGAAGATGAAGAAGAAGAAGAAGAAGAA**	-	150	0.48
Pf3D7_03_v3	CTATGTTAGTAAAAGTAAGACCTATGTTAG	-	45	0.16
Pf3D7_03_v3	ATAAATGTGGAGATAATAAATGTGGAGATA	+	90	0.48
Pf3D7_03_v3	AATTAATTAAAATATAATAAATATTATATA	-	45	<0.04
Pf3D7_03_v3	**GAAAATTTCGATTTTGAAAAATTTT**	+	25	0.08
Pf3D7_04_v3	CTATCTAAGGTCTTAATTTGACTAACATAG	+	60	0.24
Pf3D7_04_v3	ATATATATATAATTTTTTTTTTTTT	+	25	<0.04
Pf3D7_04_v3	TAAAAAAAAAAAAAAAAAAAAAAAA	+	25	<0.04
Pf3D7_06_v3	CAGTGGCACTACTACCAGCAGCACGTATAA	-	60	0.32
Pf3D7_06_v3	TTAATATTAAATAATATTAATCATATATTA	+	45	0.04
Pf3D7_06_v3	**AGGAGAAGACGAAGAAGAAGACGAAGAAGA**	-	120	0.4
Pf3D7_06_v3	ATAAGATAAAAAAAAAAAAAAAAAA	-	25	0.04
Pf3D7_06_v3	TTTTTTTTTTTTTTTTTTTTTTTTT	-	25	<0.04
Pf3D7_06_v3	CCTTTGACTTCCGTATCATTTTCTTCTTTA	+	60	0.36
Pf3D7_06_v3	TAGGTCTTACTTTCACTAACACAGGTCTTA	-	60	0.12
Pf3D7_08_v3	AGTGGAAATAAAATAAGTGGAAAGATAAAT	-	45	0.44
Pf3D7_08_v3	GTTTTTTGGATTTATTAAGTAAAGA	+	25	0.28
Pf3D7_08_v3	**AGAAGAAGAAAAAGAAGAGAAAAAAGATAA**	+	45	0.4
Pf3D7_08_v3	**AGAAGATGATGAAGAAGATGAAGATGAAGG**	-	120	0.48
Pf3D7_08_v3	CAAAATTTATATTTTTATTTTAATTTTTAT	+	45	0.04
Pf3D7_11_v3	**ATGAAAAGTAAGAAGATAAGAGTAAGTAAG**	-	60	0.48
Pf3D7_11_v3	GTCAATGCACATATCATTCCATTCCAGATA	-	60	0.44
Pf3D7_11_v3	TTTTTTAAAACTCTTTTAATTAATAAACAA	-	60	0.12
Pf3D7_11_v3	AAAAAAAAAAAAAAAAAAAAAAAAAAAAAA	+	60	0.04
Pf3D7_12_v3	AAAAAAAAAAAAAAAAAATCAGATT	-	25	<0.04
Pf3D7_12_v3	TTTTATAATGACTTTCTTTATTTAT	-	25	0.04
Pf3D7_12_v3	AAAAAAAAAAAAAAAAAAAAAAAAAAAAAT	-	45	<0.04
Pf3D7_13_v3	TCTTGAAGGAGTAAGTCCTCATAGGGTTCC	-	45	0.44
Pf3D7_13_v3	TATATTTTGTTACCATACTGTTTATTTATT	-	30	0.12
Pf3D7_13_v3	AAAAAAAAAAAAAGTAAAAACGGCATAATA	-	30	0.12

^a^ Only the first 30 nt are shown when sequence is longer.

### Association between G4FS, gene-related features and regulatory regions

**Fig 2:**
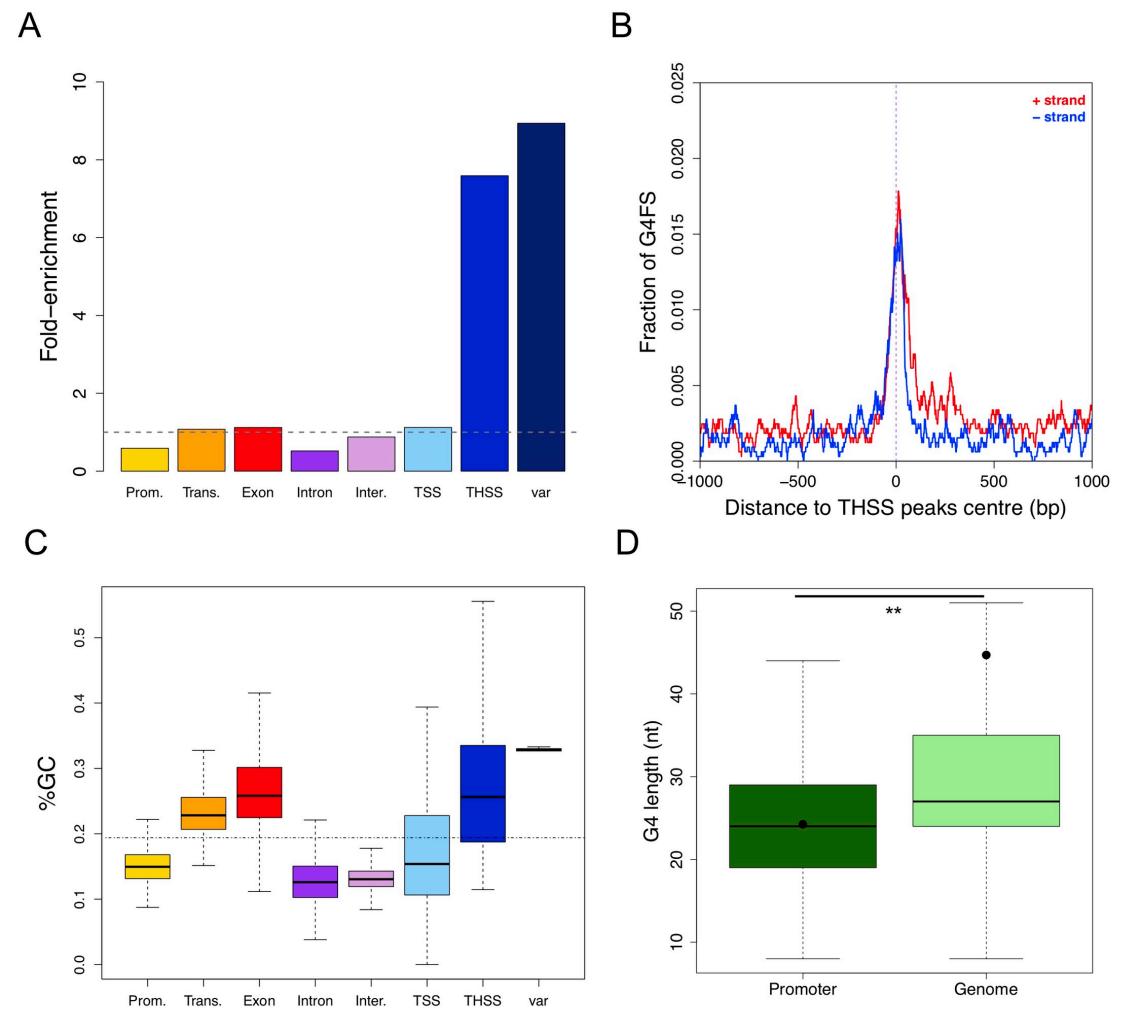
Association of G4FS with genome features of *P*. *falciparum*. (A) Fold-enrichment of G4FS in different genome features compared to 1,000 shuffling of G4H1.2 list. The dotted line indicates the fold-enrichment for the whole *P*. *falciparum* genome (value = 1.00). TSS: transcription start site. THSS: Tn5 hypersensitive sites. (B) Distribution profile of G4 motifs at threshold 1.2 around THSS peaks. The x-axis is centred on THSS peaks centre ± 1,000 bp. The red and blue lines correspond to G4FS on the coding and non-coding strands, respectively. (C) Boxplot representing the GC content in the different genomic features. The dotted line indicates the mean GC content of the *P*. *falciparum* genome. (D) Boxplot showing the G4 sequence length within promoters and in the whole genome. The mean sequence length (black point) of G4 within promoters was found significantly different from all G4 in the genome (two-tailed *t*-test, **p-value<0.01).

To investigate the putative function of G4FS in gene regulation, we examined the distribution of putative G4FS in various genomic regions, using the following genome features: promoter (defined as 2 kb upstream of the start codon), transcript, exon, intron, intergenic region (including telomeres and subtelomeres) and two regulatory regions that include transcription start sites (TSS) [50] and nucleosome-depleted regions (Tn5 hypersensitive sites, THSS) [49] (see Methods section). At all tested thresholds, a high G4FS density was observed in THSS regulatory regions (Table 1). Indeed, these regions were characterised by a strong and significant G4 enrichment (16-fold at G4H1.75) whatever the threshold used, compared to a null distribution obtained by reshuffling each G4Hunter hit list 1,000 times (Fig 2A and S3 Table). Interestingly, at a threshold of 1.2 the distribution profile of G4FS within 1 kb of THSS revealed a restricted and symmetric enrichment at the site of THSS on both strands (Fig 2B).

In *Plasmodium*, telomeres are composed of repetitions in tandem arrays of a degenerate heptamer (*i*.*e*., GGGTT(T/C)A) over 1.2 kb mean length [52]. They were shown to form stable G4s and are targeted by G4 ligands [52]. We found 98 G4FS in the telomeres (at threshold 1.2) with an average G4 length of 287 nt. It is noteworthy that, as these regions are long and highly repetitive, this low number of G4FS (98) may correspond to multiple adjacent G4s. Also, we noted the presence of 168 G4FS in subtelomeric regions composed of large non-coding regions of highly repetitive motifs, which precede the virulence multi-gene families (e.g. *var*, *rif* and *stevor* genes) prone to expansion and diversification through recombination [4].

Analysis of gene-related features revealed that the exon regions exhibit a modest but significant enrichment of G4FS at a threshold of 1.2, while the promoter and intron regions are depleted in G4FS (Fig 2A and S3 Table). Furthermore, unlike the GC poor *Dictyostelium* genome [47], the G4 enrichment correlates with the relative GC content in various *Plasmodium* genome features. For instance, *var* genes exhibit the highest GC content (33%) and are associated with the highest G4 fold-enrichment, while promoters and introns have a GC content lower than genome average, which coincides with G4 depletion (Fig 2C and S3 Table).

G4FS were not significantly enriched in TSS regions at a threshold of 1.2 (1.1 fold; Fig 2A and S3 Table). However, the local profile within 1 kb of the TSS revealed an asymmetric G4 distribution between coding and non-coding strands. Here we detected a first narrow peak on the non-coding strand and two peaks in the first 200 bp downstream the TSS (S3 Fig). This coincides with previous observations in *P*. *falciparum* describing a strand-biased G-richness at about 150 bp and 210 bp downstream of the TSS, which corresponds to a dip in nucleosome occupancy [50].

We further looked into the 288 G4FS that were located in the promoter region at threshold 1.2 (Table 1). To establish the list of genes with a predicted G4 in their promoter region (henceforth called G4-promoter genes), we computed for each G4FS from G4H1.2 list its distance to the start of the nearest coding sequence. Twenty of the 288 G4FS were discarded as they were located in telomeric repeats and/or assigned to a promoter of non-protein coding RNA or pseudogenes. The remaining 268 G4FS were assigned to 247 different promoters (S4 Table). Nineteen promoters contained 2 G4FS and the promoter of PF3D7_0308600 gene contained 3 G4FS (S4 Table). Interestingly, we observed a high enrichment of virulence genes among the G4-promoter genes (Bonferroni adjusted *p*-value = 2.47e-11): thirty-three G4FS were found in the promoter of 26 *var* genes, with 7 genes having 2 G4FS (S4 Table). Except for *var* genes, for which sense and antisense transcription initiation events were detected within the intron [50], most G4FS-associated promoters (162/268) were found at a distance <1kb from the TSS (S4 Table). Further analysis of G4 length showed that G4-promoter genes are significantly shorter than G4FS in the whole genome (Fig 2D) and numerous sequences contain multiple G-runs or long G-tracks (S4 Table), which might result in higher thermodynamically stable G4 structures [41].

### Distribution of G4FS in *var* promoter and within *var* genes

Having previously shown that G4FS co-localize with the *var* gene superfamily (Fig 1A), we computed the fold-enrichment of G4FS in 60 *var* members. The results revealed that G4FS were enriched 9-fold and 18-fold for G4H1.2 and G4H1.75, respectively, compared to G4 at positions randomly shuffled (Fig 2A and S3 Table). We found that, on average, each *var* gene contained six G4FS, of which at least one was located on the coding sequence of the first exon (S5 Table). Regarding strand orientation, we noticed a pronounced non-coding strand preference in the *var* exons (S4 Fig).

**Fig 3:**
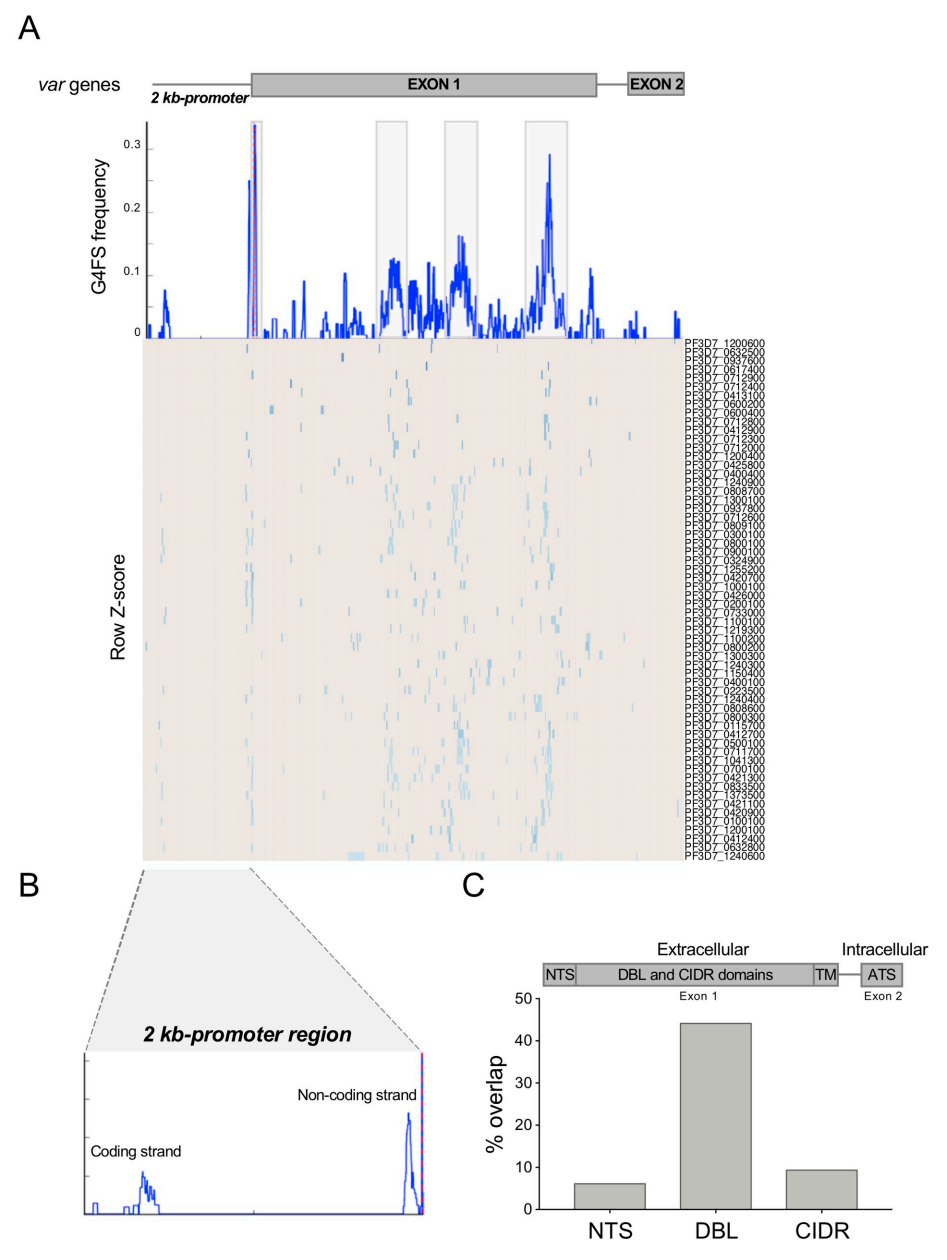
G4FS distribution within *var* genes in *P*. *falciparum*. (A) Metagene plot of G4FS frequency in the *var* gene repertoire (middle panel) that displays a two-exon structure (upper panel). The 2-kb promoter region is delimited by dotted red line. The heat map illustrates G4 positions for each gene, from which metagene plot was built (lower panel). The grey boxes represent the four intragenic G4 clusters with high G4FS frequency. (B) Magnified view of the promoter region showing two conserved loci where G4FS are found on either strand. (C) Percent overlap of G4FS with the *var* exon 1 encoded N-terminal segment (NTS), Duffy binding-like (DBL) domains and Cys-rich inter-domain regions (CIDR). For each region, the total number of G4FS overlapping to the region intervals with a minimum overlap of 1 nt was calculated. A schematic presentation of the four major PfEMP1 domains is presented on top. TM: transmembrane segment; ATS: acidic terminal segment.

Despite differences in the length and sequence of the different *var* genes, the overall gene structure is conserved amongst the family members. We therefore built a metagene plot to explore high level distribution of G4s at a threshold of 1.2 in the *var* loci (see Methods section and Fig 3A).

The metagene plot revealed two G4 clusters at the promoter level: one located on the non-coding strand, immediately upstream the ATG, harbouring 17 G4FS and a second cluster on the coding strand at ~1.6 kb from the start codon with 15 G4FS (Fig 3B and S4 Table). *Var* upstream regions (ups) are split into different categories based on their sequence and chromosomal location [39]. Most promoter G4FS were found to be present in upsB genes (S4 Table), which consists of telomeric *var* genes that are transcribed towards the centromere, in agreement with Smargiasso *et al*. [29]. Additionally, we found four G4 clusters with high G4FS frequency: one was located on the start codon in the coding strand, while the other three clusters were within exon 1 and mainly on the non-coding strand (Fig 3A and S4 Fig).

Lastly, we examined whether this G4FS cluster pattern was associated with any of the PfEMP1 protein subdomains. While the conserved exon 2 encodes an intracellular acidic terminal segment, exon 1 encodes a transmembrane domain and a polymorphic domain which is fully exposed on the surface of the RBC. The latter is composed of three types of sub-domains–a short N terminal segment (NTS) at the outermost region, a highly variable Duffy-binding-like (DBL) domain and one or two cysteine-rich interdomain regions (CIDRs) [10]. Less than 10% of G4FS overlapped with the NTS and CIDR regions (Fig 3C). However, 44% of the G4FS were present in the DBL domains—key adhesive modules of PfEMP1 involved in cytoadherence of the infected RBC to a variety of host receptors [53].

### *Var*-associated G-quadruplexes are evolutionary conserved

The human pathogen *P*. *falciparum* is most closely related to a group of parasites known as the *Laverania* subgenus, which includes *Plasmodium adleri*, *Plasmodium billcollinsi*, *Plasmodium blacklocki*, *Plasmodium reichenowi* and *Plasmodium praefalciparum* [54]. All these species naturally infect African Great Apes (and not humans) but share a common ancestor with *P*. *falciparum* [54]. We therefore looked at the G4FS distribution in the five genomes using G4Hunter as described above for *P*. *falciparum*. All genomes have similar AT content (from 80.9 to 81.5%).

**Fig 4:**
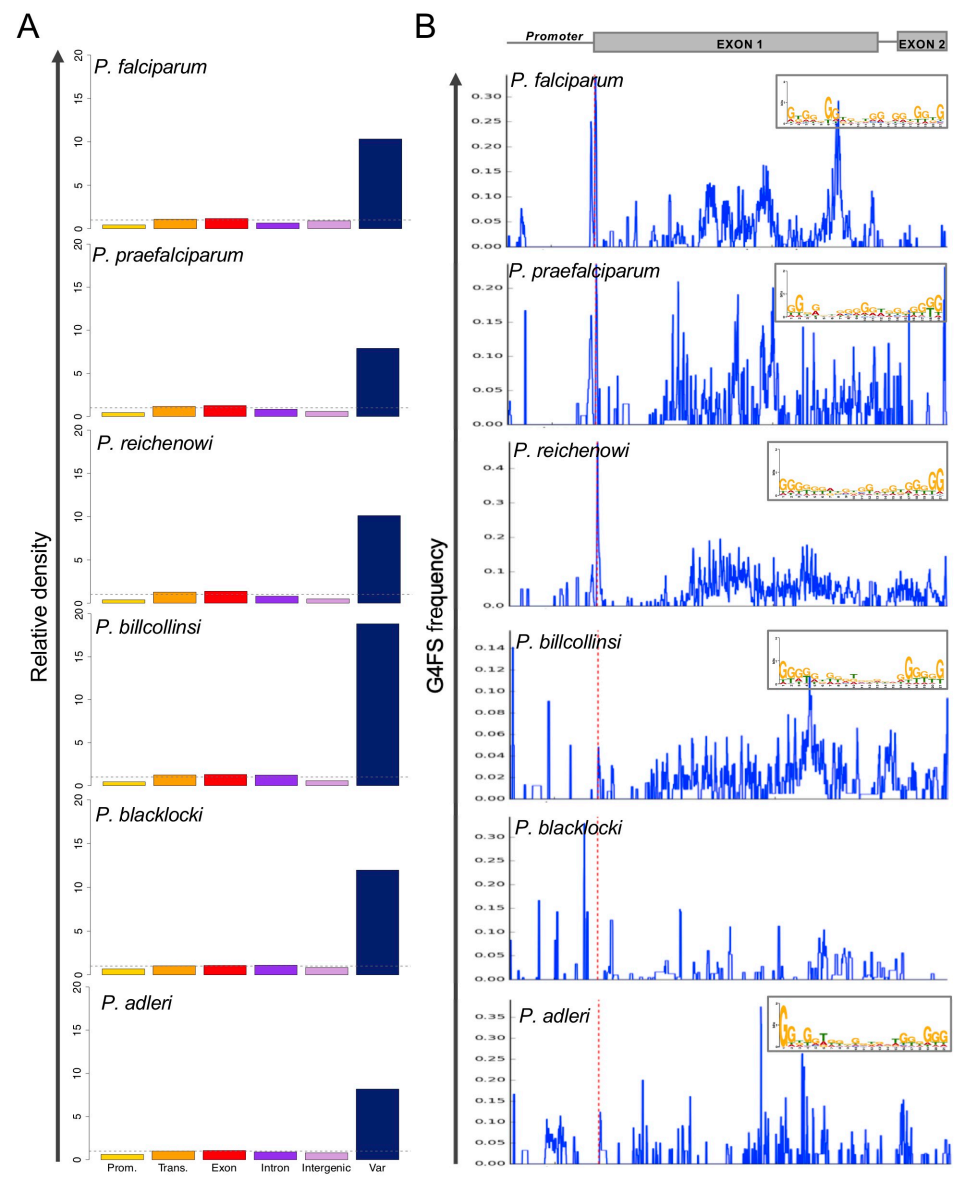
G4FS distribution in *Laverania* species. (A) Relative density of G4FS for different genome features of various *Plasmodium* species from *Laverania* subgenus (G4Hunter, threshold 1.2). *Plasmodium* species are sorted according to their genetic distance from *P*. *falciparum* with the closest species on top. G4FS presented here are the results of G4Hunter analysis after applying a threshold of 1.2. (B) Metagene profiles of G4FS in *var* genes of *Laverania* species. The x-axis represents upstream (2 kb) and transcript region (delimited by dotted red line). G4FS frequency is plotted on y-axis. The most significant conserved motifs identified with MEME software [55] are illustrated on the upper right panel. No conserved motif was found for *P*. *blacklocki*.

G4FS distribution at a threshold of 1.2 revealed a chromosome-bias on chromosome 4 for all species analysed, as observed for *P*. *falciparum* (S5 Fig). Furthermore, *P*. *blacklocki* presented a ~2-fold lower G4FS density than *P*. *falciparum*, while *P*. *reichenowi* had a G4FS density close to *P*. *falciparum* (Table 1). Association of G4FS with gene-related features (*i*.*e*., promoter, transcript, exon, intron) unveiled an evolutionary conserved pattern of enrichment in all features with *var* genes accounting for the highest enrichment (Fig 4A), suggesting that G4 motifs were positively selected in these genes throughout evolution. Metagene profiles were built for the *Laverania var* genes and revealed intriguing differences among the species. In contrast to *P*. *blacklocki* and *P*. *adleri* that are more evolutionarily distant from *P*. *falciparum* [54], *P*. *praefalciparum*, *P*. *reichenowi* and *P*. *billcollinsi* showed intragenic clustering of G4FS comparable to *P*. *falciparum* (Fig 4B). We analysed the sequence conservation of the intragenic G4FS and a G-rich motif was found in all species but *P*. *blacklocki* (Fig 4B upper right panel). These results highlight the evolutionary conservation of G4FS at gene and sequence levels, within *var* genes across closely related *Plasmodium* species.

### G4FS are associated with recombination events in subtelomeric regions

**Fig 5:**
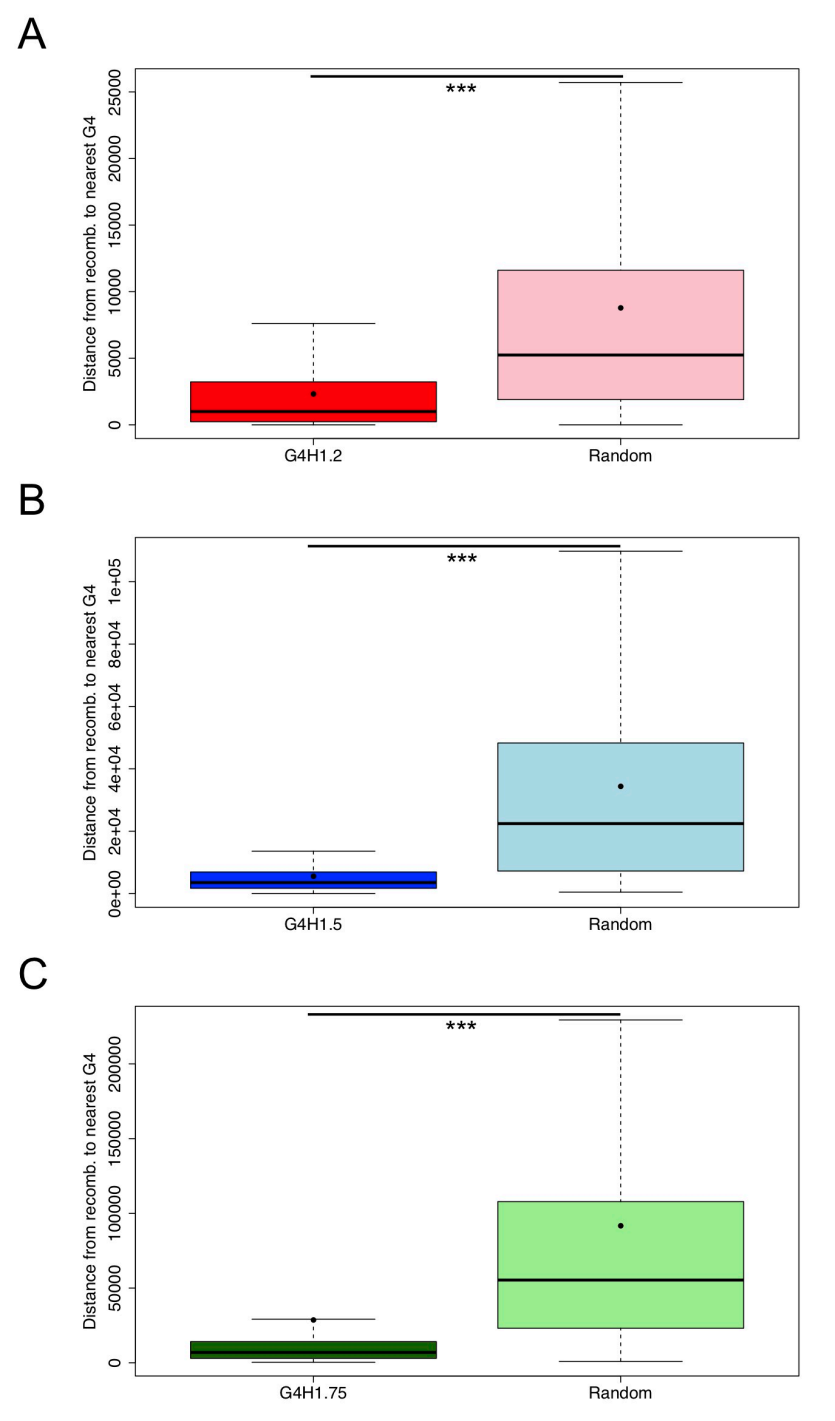
G4FS are associated to recombination events. (A-C) Boxplots showing the distance from recombination breakpoints to the nearest G4FS for the three applied thresholds: G4H1.2 (A), G4H1.5 (B) and G4H1.75 (C). For comparison, a null dataset with 10 randomizations of recombination sites was generated and one random condition is shown for each threshold. The mean distance to recombination breakpoints of each G4H list (black point) was found significantly different from null dataset (Wilcoxon test, ***p-value <0.001).

The presence of G-quadruplexes has been reported to induce genome instability *i*.*e*. higher than normal rates of mutation, in yeast [56] and human cells [16]. While diseases such as cancer can be a consequence of genome instability, for pathogens like *Plasmodium* this could lead to antigen diversification and would thus be advantageous for host immune evasion. We therefore assessed the distance from each recombination hotspot [40] defined with a width of less than 10 nt (n = 168) to its nearest G4FS. For comparison, a null dataset of breakpoint positions randomly shuffled 10 times across the genome was generated and the distances to their nearest G4FS were determined for three different G4Hunter thresholds. The mean distances from each G4FS to a recombination breakpoint were 2.3 kb versus 9.2 kb for the null dataset, 5.5 kb versus 33.8 kb, and 28.6 kb versus 101.8 kb for G4H1.2 (Fig 5A), G4H1.5 (Fig 5B) and G4H1.75 (Fig 5C), respectively. In all conditions, the difference between the G4 dataset and the null dataset was found to be highly significant (Wilcoxon test ***p-value <0.001). These results show a much stronger link between G4FS and recombination hotspots than what was previously reported by Stanton *et al*. [40], where the mean distance to the nearest G4 was far larger (133 kb). These findings point out to a putative role of the G4FS clustering pattern, observed within the *var* gene regions that encode the, surface exposed, submodules of PfEMP1.

### Validation of G4 formation *in vitro* of G4FS candidates

**Fig 6:**
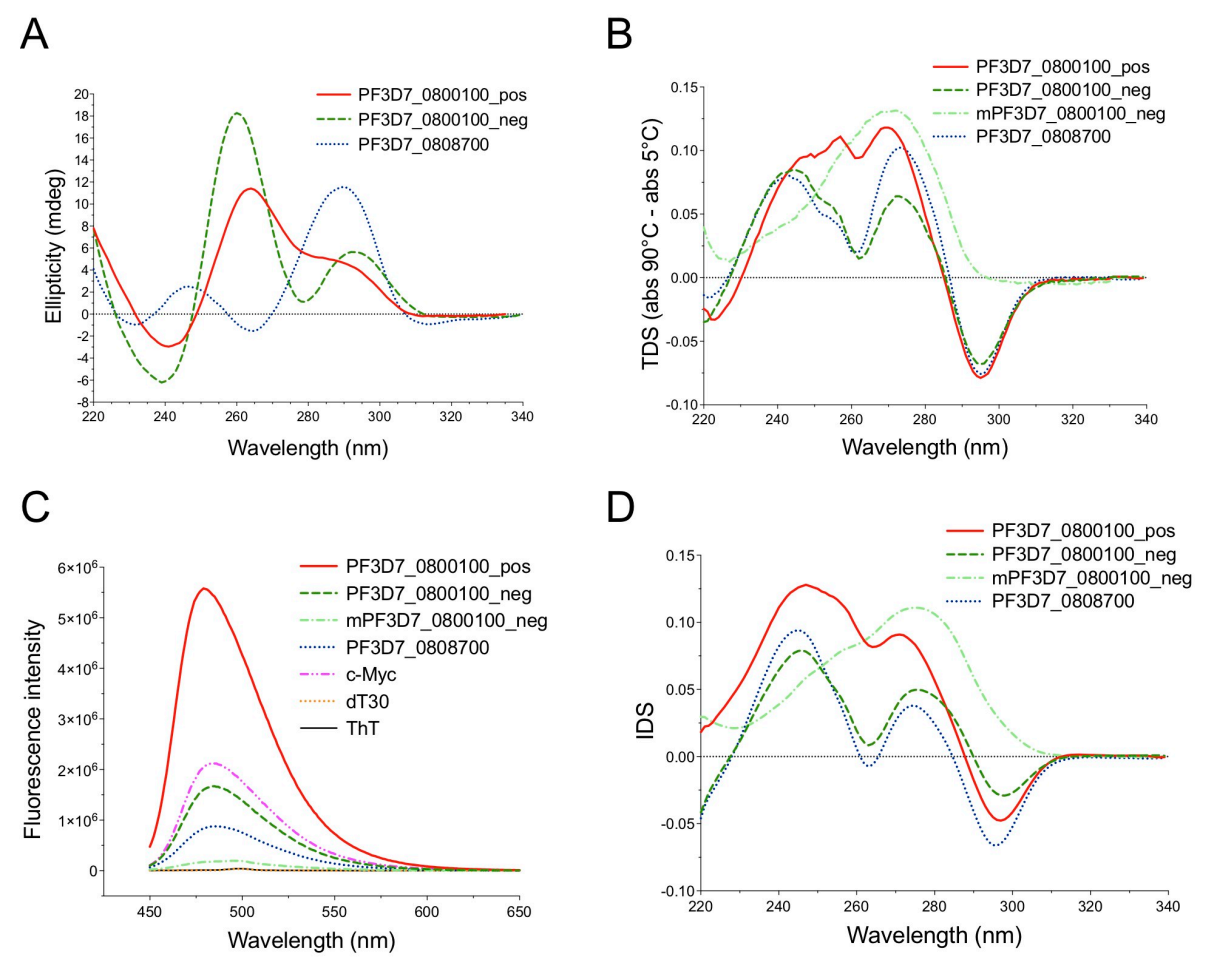
Biophysical characterization and transcriptional control of G4FS in *var* promoters. (A) Circular dichroism (CD) spectra of the three selected G4FS in *var* promoters. The measurements were carried out in 100 mM KCl at 6 μM and at 5°C, except PF3D7_0800100_pos which was folded at 3 μM and at 20°C. The G4 oligonucleotide sequences are presented in Table 3. (B) Thermal difference spectra (TDS) of the three G4FS and mPF3D7_0800100_neg, the mutated version of PF3D7_0800100_neg, in 100 mM KCl and at 4 μM. (C) Fluorescence emission spectra of ThT in the presence of selected G4FS. DNA samples were prepared at 1 μM in 100 mM KCl and incubated with 0.5 μM ThT. The c-Myc and dT30 sequences (Table 3) were used as positive and negative controls, respectively. (D) Isothermal difference spectra (IDS) of the G4FS at 20°C. The DNA samples were folded in 100 mM KCl at 4 μM.

The widespread presence of G4FS within the promoter of *var* genes led us to hypothesise that, when structured, these could have an impact on the transcription of these genes. To verify that we first validated three promoter G4FS as *bona fide* G4s using standard spectroscopic assays. Two G4FS were present in the promoter of the *var* PF3D7_0800100. The first sequence (PF3D7_0800100_pos) is located at 1.6 kb from the start codon on the coding strand (S4 Table), and contains 4 G-runs of at least 3G, that are linked by relatively short loops (up to 10 nt) (Table 3). This G4FS is conserved among 12 *var* promoters and has also been predicted to form a G4 by others, using QGRS Mapper [29,40]. The second sequence (PF3D7_0800100_neg) was not identified in previous studies and is located on the non-coding strand, 34 bp upstream of the start codon (S4 Table). Its DNA sequence is predictive of a 2-quartet conformation with 7 G-runs of at least 2G (Table 3). A third G4FS (PF3D7_0808700), located on the opposite strand 76 bp upstream the start codon of the *var* gene PF3D7_0808700 (S4 Table), is conserved among 6 *var* promoters and contains several 2G-tracks (Table 3). For each G4FS, the G4Hunter score is shown in Table 3. For the two G4FS in PF3D7_0800100, the circular dichroism (CD) spectra exhibited similar shapes with two positive peaks at 263 and 293 nm and a negative peak at 240 nm (Fig 6A). These CD spectra showed characteristics of both parallel and anti-parallel G4 structures [57]. In contrast, the CD spectra of PF3D7_0808700 displayed a positive ellipticity peak at 290 nm and a negative peak at 264 nm (Fig 6A), suggesting the formation of anti-parallel G4 [57]. Additionally, thermal difference spectra (TDS) were defined by two positive peaks at around 240 and 270 nm, and a negative peak at 295 nm (Fig 6B), which are indicative of G4 formation [58]. G4 formation was also confirmed by a fluorescence light-up assay using Thioflavin T (ThT) (Table 3) [59]. As shown in Fig 6C, the ThT fluorescence intensity increased in presence of the selected G4FS, compared to the emission spectra of ThT alone. By determining the ratio of fluorescence intensity in presence of G4FS by the background fluorescence (FI/FI_0_), we observed that the G4FS led to an increase of at least 40-fold compared to ThT alone, in agreement with G4 formation (S6 Fig). In addition, we recorded isothermal difference spectra (IDS) for further confirmation. The IDS spectra showed negative peaks at 295 nm and positive peaks at 273 nm that are characteristic of G4 formation (Fig 6D). Finally, we recorded the melting curves at 295 nm. They both presented an inverted sigmoid shape which is typical of G4 structures (S7 Fig). The deduced melting temperatures (*T*_m_) for PF3D7_0800100_pos and PF3D7_0808700 were 59 ± 1°C and 57 ± 1°C, respectively, thus confirming the high stability of the G4s [29], while the *T*_m_ for the 2-quartet motif PF3D7_0800100_neg was 46 ± 1°C. Overall, these results demonstrate that each of these G4FS does form stable G-quadruplex structures *in vitro*, under near-physiological conditions.

**Table 3:** Sequence of G4 oligonucleotides used in this study, with the strand position and the G4Hunter score. The two oligonucleotides c-Myc and dT30 were used as positive and negative controls, respectively, for ThT assay.

G4 name	G4 sequence	Strand	Length	G4Hunter score
PF3D7_0800100_pos	**GGG**TTAA**GGG**TATAACTTTA**GGGG**TTA**GGG**	+	30	1.32
PF3D7_0800100_neg	GT**GG**TA**GG**TGT**GGG**C**GG**T**GG**T**GG**T**GG**CG	-	28	1.32
mPF3D7_0800100_neg	GTC**G**TAC**G**TGT**G**T**G**CT**G**TA**G**TT**G**TA**G**CG	-	28	0.25
PF3D7_0808700	**GG**T**GG**TGT**GG**TGT**GG**TGT**GGGG**TG	-	24	1.5
PF3D7_0704800	**GG**CAA**GGG**TA**GGGG**A**GG**AGAGAAAGAGAGAG	+	31	1.4
PF3D7_1121300	GT**GGGGGG**AGT**GG**ATT**GG**TTTG	+	22	1.4
PF3D7_0613300	GAATGAGA**GGG**TT**GGGG**AGTA**GG**AAACAAATC**GG**	+	34	1.32
PF3D7_0613300-no-tail	**GGG**TT**GGGG**AGTA**GG**AAACAAATC**GG**	+	26	1.23
c-Myc	TGA**GGG**T**GGG**TA**GGG**T**GGG**TAA	NA	22	1.68
dT30	TTTTTTTTTTTTTTTTTTTTTTTTTTTTTT	NA	30	0

### Stabilization of a G-quadruplex in an ectopic *var* promoter represses gene expression

**Fig 7:**
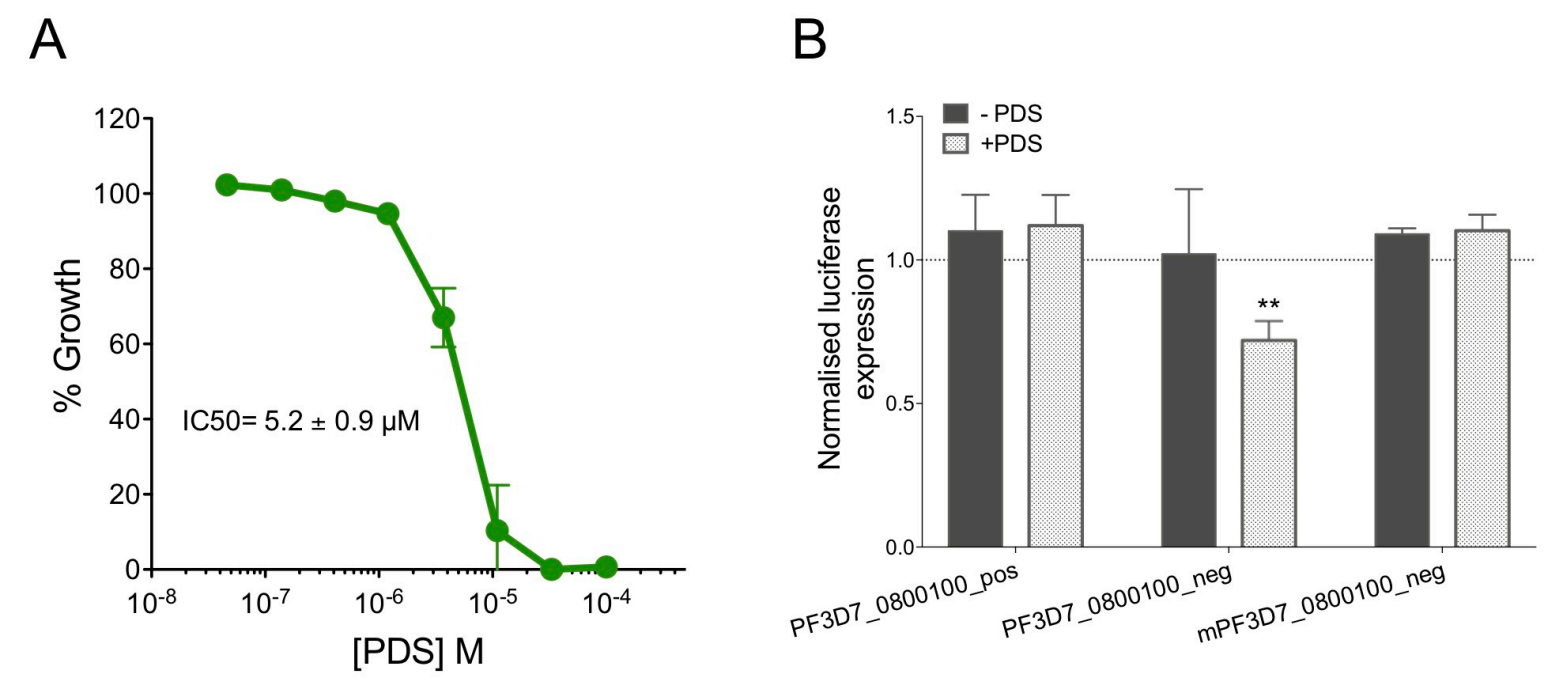
Transcriptional control of G4FS in *var* promoters. (A) Growth curve analysis and IC_50_ determination of *P*. *falciparum* 3D7 strain treated with PDS using Desjardins experiment [62]. Error bars represent standard deviations of experiments done in triplicate. (B) Promoter-driven luciferase assay performed by transient transfection of *P*. *falciparum* at ring stage (dark grey bars) with plasmids that encode the *luciferase* gene under *calmodulin* gene promoter with the G4 sequence that was cloned upstream the promoter. Parasites were maintained for 48h before saponin-lysis and luciferase signal measurement. In parallel, parasites were treated with 1 μM pyridostatin (PDS) (light grey bars). Results are the mean of three independent experiments (two-tailed *t*-test, **p-value<0.01).

Several studies have shown that *var* gene expression is regulated by multi-layered mechanisms that are still not yet fully elucidated. Given the presence of *bona fide* G4s in the promoter of these genes we hypothesized that these might contribute to this complex process. We therefore studied the impact of the highly selective G4-ligand pyridostatin (PDS) on *var* gene expression by qPCR. PDS stabilizes G4s and was shown to deregulate gene expression when targeting G4-containing promoters in human cells [16,60]. As PDS is toxic for *P*. *falciparum* (IC_50_ = 5.2 ± 0.9 μM; Fig 7A), we chose a compound concentration (1 μM) that did not affect cell growth. For that we measured *var* expression levels in ring-stage parasites, when *var* expression is maximal [4], using gene-specific primers in a qPCR assay (see Methods section). However, at 58h post-treatment (*i*.*e*., second-cycle rings), the expression patterns in treated and untreated conditions remained similar in the presence of PDS (S8 Fig).

To exclude a potential dampening effect imposed by the native environment and the above mentioned multi-layered regulatory programme, we decided to assess the potential of the promoter-G4s to modulate expression in an ectopic setting. We thus assayed the impact on transcription of the presence of two of the validated G4 motifs (PF3D7_0800100_pos and PF3D7_0800100_neg) through a promoter-driven luciferase assay. After several attempts with the endogenous *var* promoter, it was difficult to detect sufficient reporter gene expression [61]. We therefore decided to use a promoter with higher transcriptional activity (*calmodulin* gene) and cloned each of the G4FS immediately upstream of the promoter sequence. *P*. *falciparum* parasites were co-transfected with a second plasmid encoding the Renilla luciferase regulated by the same *calmodulin* promoter to serve as an internal control. While none of the G4s led to reporter gene modulation under normal conditions (Fig 7B), we observed a significant decrease of Firefly luciferase expression with PF3D7_0800100_neg in the presence of PDS (Fig 7B). As a negative control we used a mutated version of this G4 sequence (mPF3D7_0800100_neg), where the introduction of specific point mutations prevented G4 folding (Table 3), as confirmed by TDS (Fig 6B), the absence of ThT fluorescence emission (Fig 6C and S6 Fig) and IDS (Fig 6D). No alteration of luciferase expression was detected in the negative control mPF3D7_0800100_neg (Fig 7B).

Both PF3D7_0800100_pos and PF3D7_0800100_neg folded into stable G-quadruplex structures, as confirmed by biophysical methods, but their strand positioning led to different levels of expression. Extracted from the native loci, the presence of the G4 structure on the non-coding strand of the ectopic reporter gene was correlated with decreased transcription, while the G4 on the coding strand did not affect gene expression. Moreover, the effect on gene regulation required the G4 stabilization by the G4 ligand PDS. Although these conditions did not reflect the genomic context, as the G4s were inserted into an exogenous promoter, these results demonstrate that PDS may target G4s *in vivo*, in *Plasmodium*.

### Pyridostatin alters genome-wide gene expression

**Fig 8:**
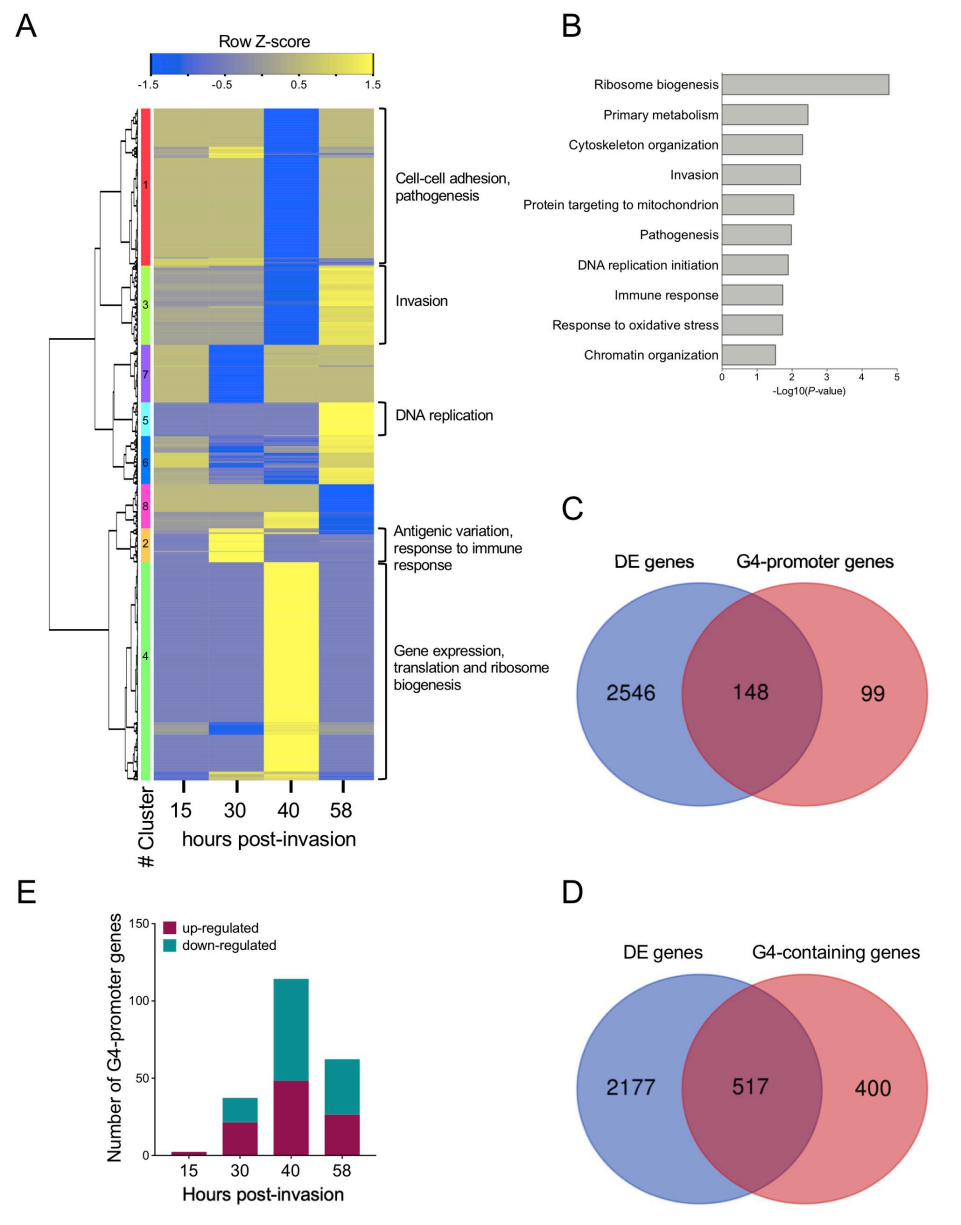
Transcriptome profiling of PDS-treated parasites. (A) Heat map of RNA-Seq data with hierarchical clustering based on gene expression profiles. Heat map colours ranging from blue to yellow indicate increasing log2 fold-change of expressed genes (rows) in PDS-treated parasites at the different time-points (columns) as compared to untreated parasites (adj. *p*-value <0.01 and fold-change >2). On the right panel, the gene ontology (GO) enrichment analysis was performed for genes within each cluster using PlasmoDB [46]. (B) GO enrichment analysis of differentially expressed genes during PDS treatment using topGO. The 10 top highly enriched GO Biological Processes categories are shown (*p*-value < 0.05). (C-D) Venn diagrams showing the number of differentially expressed (DE) genes that overlap with G4-promoter genes (C) and G4-containing genes (D). (E) Number of up- and down-regulated genes that contain a G4 in their promoter at the different time-points.

Although the IC_50_ of PDS was 5.2 ± 0.9 μM (Fig 7A), we found that only at a concentration of 0.6 μM were the parasites able to maintain their synchronicity for two cycles. To assess the impact of G4 stabilization on the transcriptome of *P*. *falciparum*, we cultured parasites with and without PDS, and harvested samples at different stages of the intra-erythrocytic cycle (ring stage at 15 hours post-invasion (hpi), trophozoite at 30 hpi, schizont at 40 hpi and ring stage at 58 hpi after reinvasion). A total of 2,694 genes were differentially expressed (DE) throughout *Plasmodium* development (adj. *p*-value <0.01 and fold-change >2; S6 Table). Strikingly, a large cohort of genes was deregulated at 40 hpi (Fig 8A and S6 Table). Comparison of both ring stages at 15 hpi and 58 hpi revealed that parasites were more affected by PDS treatment after a complete 48 h-cycle, suggesting that PDS effects tend to accumulate with longer exposure times (Fig 8A). The gene ontology (GO) enrichment analysis indicated that several metabolic pathways related to DNA biology, such as DNA replication, gene expression and translation (all of which G4-related pathways, were profoundly affected by the PDS treatment (Fig 8A and 8B). Specifically, we observed the modulation of the expression of half (13/27) of the putative members of the Apicomplexan AP2 (ApiAP2) family of transcription factors [63], which might justify the extent of gene deregulation detected (S6 Table). GO analysis has also highlighted an important up-regulation at the schizont stage (40 hpi) of genes involved in ribosome biogenesis (Fig 8B). Genes involved in virulence and antigenic variation, which usually have their peak of expression at ring stage (*i*.*e*., up to ~15 hpi), were up-regulated later in the cycle, in trophozoites (30 hpi) (Fig 8A); this included half of the *var* gene repertoire (31/~60). Interestingly, the *var* gene PF3D7_0800100 was downregulated at 58 hpi (log2FC = -1.44; p-value: 1.2E^-10^) in agreement with our reporter assay findings. We then intersected the DE genes with our set of G4-promoter genes (n = 247) and G4-containing genes (n = 917) with at least one G4 in coding sequence (threshold 1.2), and we found that 60% of G4-promoter genes and 56% of G4-containing genes were de-regulated upon PDS treatment (Fig 8C and 8D). Among them, 23 of the promoter G4s and 44 of the intragenic G4s were identified by the G4-seq method [31], meaning that 21% of the G4s found by G4-seq are associated to gene deregulation following PDS treatment in *P*. *falciparum*. The expression profile of G4-promoter genes showed that most genes were deregulated at 40 hpi (Fig 8E).

**Fig 9:**
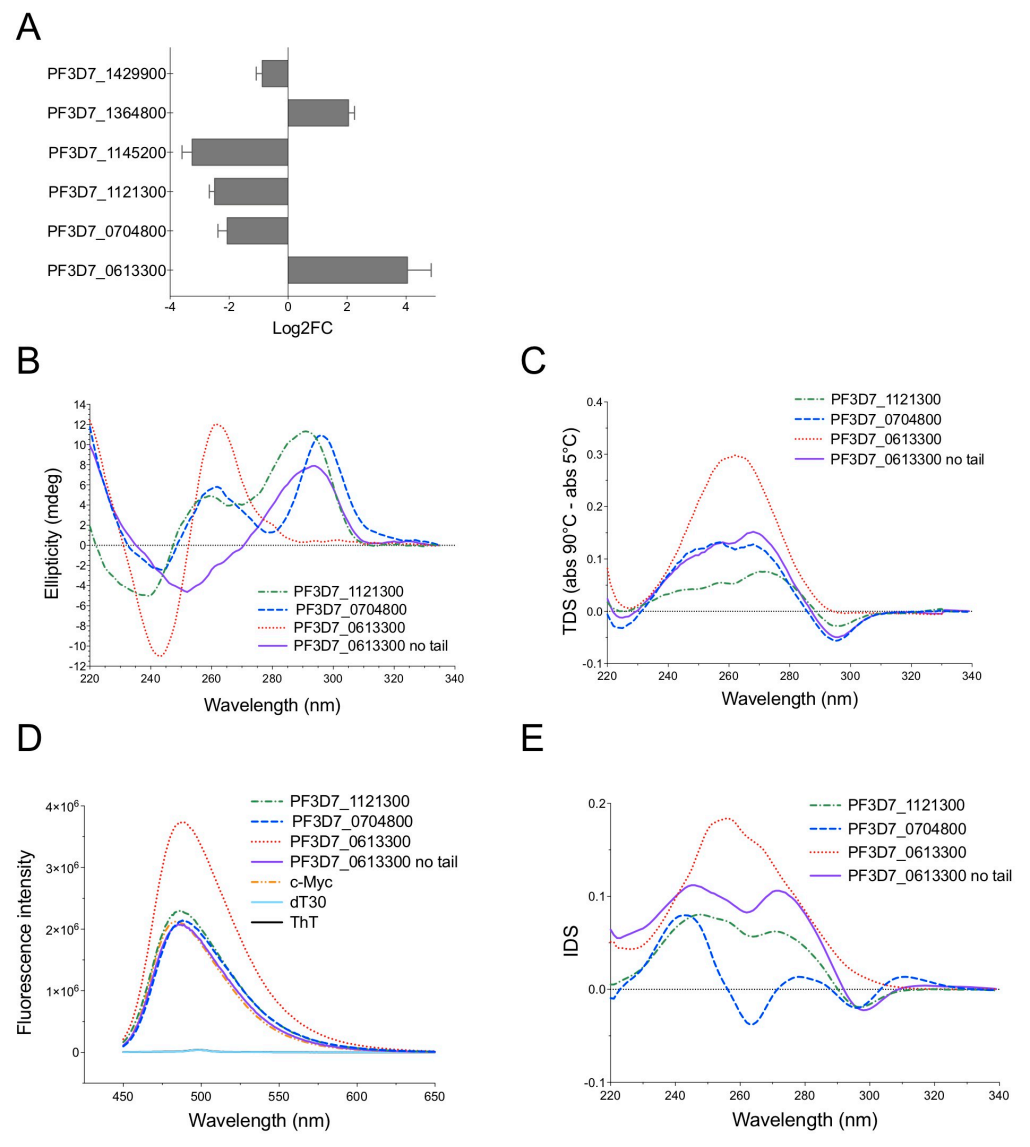
Validation of RNA-Seq data by qRT-PCR and biophysical characterization of selected G4FS. (A) Relative expression of selected target genes expressed in log2 Fold-change (Log2FC). Error bars correspond to the standard deviation of the ΔΔC_T_ value. (B) CD spectra of four promoter G4FS. For PF3D7_0613300-no-tail, an 8-nt 5’ tail has been removed. The G4 oligonucleotide sequences are shown in Table 3. (C) Thermal difference spectra (TDS) of selected G4FS. For CD and TDS, DNA samples were folded at 20°C in 100 mM KCl at 6 μM, except PF3D7_0613300 with and without tail, which have been folded at 3 μM at 5°C. (D) Fluorescence emission spectra of ThT in the presence of selected G4FS. The c-Myc and dT30 sequences were used as positive and negative controls, respectively. (E) Isothermal difference spectra (IDS) of the four promoter G4FS.

To validate our RNA-Seq results, we selected six G4-promoter genes amongst the DE genes to be assessed by qRT-PCR: PF3D7_1121300 at 30 hpi, PF3D7_0704800, PF3D7_1145200, PF3D7_1364800 and PF3D7_1429900 at 40 hpi (ADP-dependent DNA helicase RecQ protein that was recently associated with genome instability in *Plasmodium* [42]) and PF3D7_0613300 at 58 hpi. All genes exhibited significant changes in a trend consistent with the RNA-Seq results (Fig 9A). We then evaluated the ability of G4FS present in the promoters of PF3D7_1121300 (30 hpi), PF3D7_0704800 (40 hpi) and PF3D7_0613300 (58 hpi) to form a G4 structure *in vitro* (Table 3). For the remaining genes, long G repeats (≥6) or incomplete G-runs (*i*.*e*., a number of G-runs <4) in G4FS rendered difficult the study of G4 folding by biophysical methods (S4 Table). The CD spectra and TDS revealed a G4 signature for PF3D7_1121300 and PF3D7_0704800 but not PF3D7_0613300 (Fig 9B and 9C) [57,58], with *T*_m_ values of 32 ± 2°C and 53 ± 1°C for PF3D7_1121300 and PF3D7_0704800, respectively. All G4FS led to an increase of fluorescence intensity following ThT incubation (Fig 9D) [59]. The IDS of PF3D7_1121300 and PF3D7_0704800 exhibited a positive peak at 273 nm and a negative peak at 295 nm that are characteristic of G4 formation, while PF3D7_0613300 did not show a specific G4 profile (Fig 9E). This sequence of 34 nt contains an 8-nt tail in its 5’ end (Table 3). We therefore analyzed the ability of a shorter sequence without the 8-nt tail (PF3D7_0613300-no-tail) to form a G4 (Table 3). Interestingly, CD spectra and TDS showed that PF3D7_0613300-no-tail does fold into G4 (Fig 9B and 9C, respectively), but has a low *T*_m_ value of 34 ± 2°C. The G4 formation of PF3D7_0613300-no-tail was further confirmed by ThT assay with an enhancement of emission fluorescence of ThT in presence of the G4FS, compared to ThT alone (Fig 9D). Finally, IDS of PF3D7_0613300-no-tail revealed a similar profile as PF3D7_1121300 and PF3D7_0704800 (Fig 9E), thus confirming the ability of the G4FS to fold into G4. Altogether, these results demonstrate that PDS deregulates a substantial proportion of the *Plasmodium* transcriptome and this effect includes G4-promoter genes.

## Discussion

The complexity of gene expression regulation in malaria parasites is still being unravelled. Emerging evidence has highlighted the importance of G-quadruplexes in gene expression regulation in eukaryotes [12,13]. In a recent landmark study, a method termed G4-seq has been employed to experimentally study the distribution of G4s in 12 species that included model organisms and pathogens of clinical relevance such as *P*. *falciparum* [31]. While its performance was optimal for %GC balanced genomes such as the human genome where the false discovery rate (FDR) was estimated to be 8%, the Illumina sequencing conditions (*e*.*g*. primer extension reaction performed at 55°C or above) and the parameters for G4 detection led this method to perform sub-optimally for the AT-rich genome of *P*. *falciparum* (~80% AT; FDR ~30%). For this reason we turned to the G4Hunter algorithm, a tool that has been extensively used for the prediction of G4s in different types of genomes, including GC poor ones [41,47], to perform the most comprehensive analysis to date of G4FS in malaria parasites. To ensure that G4Hunter was indeed suitable to study the *P*. *falciparum* genome we undertook different comparison tests between our dataset and the one generated using the G4-seq method [31], and showed that former outperformed the latter in various instances. As biophysical validation experiments have demonstrated G4Hunter’s accuracy at thresholds, typically between 1.2 and 2.0, we looked at G4s predicted with a high threshold (1.75), that were not found by G4-seq (108/145), and stable G4 formation was highly likely for most of them. Furthermore, we showed in our study that out of the five *P*. *falciparum* G4 sequences that were confirmed to form a stable G4, four were not detected by G4-seq hence indicating that G4-seq likely missed these hits.

Here we have shown that the genomes of *Plasmodium* spp. display an unusual distribution of putative G4FS. Indeed, the *P*. *falciparum* genome is characterized not only by a particularly low G4FS density, which is 26-fold lower than the human genome, but also an atypical genomic distribution. For instance, while nucleosome-depleted regions are significantly enriched in G4FS, likely due to the fact that these regions define chromatin accessibility sites, promoters and introns are depleted. This contrasts to other organisms, where G4 motifs are enriched in the first intron and in promoters, as is the case of the human genome [41,64,65], and could be a consequence of the near ~90% AT-richness of the *Plasmodium* intergenic regions [39]. Furthermore, although no enrichment was detected in TSS regions, the local profile within 1 kb of the TSS revealed an asymmetric G4 distribution between coding and non-coding strands which may influence transcription levels or mRNA stability [66,67].

A set of genes displayed a significant enrichment in G4FS—the *var* multi-gene family, which encodes the major virulence factor in *Plasmodium*, in agreement with previous studies [29,40]. We showed that G4FS were enriched in the promoter and in the first exon with an over-representation on the reverse strand, a pattern that was evolutionarily conserved in related species of *Plasmodium*. Several of these promoter G4FS were validated using standard spectroscopic assays, thus confirming the accuracy of G4Hunter predictions. Gene regulation by G4 stabilization has been poorly studied in *Plasmodium*. Some studies have used a limited set of genes, including *rifin* and *var* genes [36,37]. However, the two *rifin* genes tested did not contain a G4 in the promoter but rather in the coding sequence [36] and no difference was observed in the level of expression when a G4 was present or not in the promoter of *var* genes [37]. Here we measured the effect of pyridostatin, a high affinity G4 ligand, on the expression of the *var* members. Using an ectopic luciferase reporter system, we showed that under stabilising conditions, the *bona fide* promoter-G4 present in the non-coding strand of the *var* gene PF3D7_0800100 led to a decrease in reporter gene expression. Modulation from the native *var* locus was more challenging to quantify by qPCR, but *var* expression de-regulation was observed by RNA-Seq, upon PDS treatment. Overall, the PDS effect was more pronounced when the G4 was extracted from the *var* native loci, *i*.*e*., into an ectopic setting. We thus suggest that G4 positioning and modulation is likely involved in the native locus *var* expression regulation, and consequently parasite virulence, but its effect is likely optimal within the context of a multi-layered process.

G-quadruplexes have been reported to induce DNA damage [16] and genome instability [56]. Accordingly, in the *P*. *falcipa*rum genome, G4FS are located in the vicinity of recombination breakpoints, in agreement with previous study [40]. We nevertheless evaluated the mean distance from G4FS to the nearest recombination breakpoints to be 5-fold to 58-fold lower than previously described [40]. Considering that *de novo* structural variations associated with intraerythrocytic mitotic events are enriched in and around *var* genes [68,69], where a high number of G4FS is present, it is thus likely that G4FS may favour recombination events within *var* genes, thus increasing the diversity of the malaria surface antigens. Indeed, the positioning of the G4FS in regions coding for highly polymorphic, surface exposed, protein subdomains, support the hypothesis of G4s likely role as antigenic diversity potentiators. Furthermore, the fact that this organisation is conserved in related *Plasmodium* species leads us to hypothesize that the evolutionary conservation of the positioning of the G4FS within *var* genes might be critical to ensure generation of antigenic diversity.

To investigate the impact of G4s on transcription, studies have most commonly focused on specific sets of genes. As transcription is pervasive in malaria parasites, in this study, we performed the first transcriptome wide analysis upon G4 stabilization via pyridostatin. Under such conditions we observed large-scale perturbations *in vivo*, which included genes both harbouring G4FS or not. Previous studies performed on human cells that were exposed to TMPyP4 or the bisquinolinium compound PhenDC3 have highlighted both direct and indirect effects of G4 stabilization on genome-wide expression changes [70,71]. Indirect effects could be the result of the downstream effect of G4-mediated gene regulation on other genes or the non-specific binding of the G4-ligand. Amongst the PDS-mediated de-regulated genes we found genes involved in ribosome biogenesis and several metabolic pathways related to DNA biology, consistent with PDS directly binding nucleic acid G4 targets. Furthermore, our DE dataset included half of the members of the ApiAP2 family of transcription factors and helicases such as RecQ [42] (log2FC = -1,3; *p*-value = 7.7E^-22^) whose downregulation has previously been shown to lead to the de-regulation of several hundred genes. These multiple associations support the hypothesis that, in malaria parasites, G4s play an important role in gene regulation either locally, by directly repressing the expression of the gene, or with genome wide repercussions through modulation of the expression of transcriptional regulators, such as transcription factors.

The present work has shed new light on the regulatory roles of G-quadruplexes in *Plasmodium* and taken us a step forward in the pursuit of druggable regulators of parasite biology.

## Methods

### G4Hunter search

Genome files for all *Plasmodium* species used in this study were downloaded in FASTA format from the PlasmoDB website (https://plasmodb.org/plasmo/) [46] (version 28 for *P*. *falciparum* 3D7 and version 36 for *Plasmodium adleri* (*P*. *adleri* G01), *Plasmodium billcollinsi* (*P*. *billcollinsi* G01), *Plasmodium blacklocki* (*P*. *blacklocki* G01), *Plasmodium reichenowi* (*P*. *reichenowi* G01) and *Plasmodium praefalciparum* (*P*. *praefalciparum* G01)). For non-*P*. *falciparum* species, as genome assembly was not complete, the contigs that were not assembled into nuclear chromosomes were not considered for the analysis. G4 search was performed on the 14 nuclear chromosomes of all *Plasmodium* species used in this study using G4Hunter, with a sliding window length of 25 nucleotides, as previously described [41]. Bioinformatics pipelines were conducted with R (v3.5.2). The two R scripts containing G4Hunter functions and bioinformatics procedures are provided as supplementary files (S1 Method and S2 Method, respectively). Putative G4 forming sequences (G4FS) were retrieved and sorted according to their G4 folding propensity, where high scores represent high likelihood of folding. We then applied different thresholds to select G4FS with scores above 1.2, 1.5 and 1.75, resulting in three G4 lists (G4H1.2, G4H1.5 and G4H1.75). Note that G4FS shorter than the starting window (25 nt) may be obtained as a result of the G4Hunter optimization procedure. For example, with a threshold of 1.2, a run of 8 consecutive G is sufficient to generate a score of 1.28 (assuming no other C and G are present) in a window of 25 nt. The optimization (trimming) part of G4Hunter keeps this 8G core. The full list of G4 sequences retrieved for each G4H score is provided in S1 Table.

The G4Hunter thresholds were chosen based on a study by Bedrat *et al*. [41], where the authors established that threshold values of 1 or 1.2 provided the highest sensitivity and specificity. Accordingly, we chose a score of 1.2 to obtain the most comprehensive G4FS lists while limiting the rate of false negatives, and a higher threshold of 1.75 to limit the number of false positives.

### Bioinformatics analysis

Gene annotation files matching the genome files were downloaded from PlasmoDB [46]. All bioinformatics procedures were conducted with R (v3.5.2) using the following packages: GenomicFeatures (v1.34.7) [72], Biostrings (v2.50.2) and rtracklayer (v1.42.2) [73]. For the fold enrichment analysis, a first feature list was created with five categories: promoter, transcript, exon, intron and intergenic regions. In annotation files, transcript genomic coordinates correspond to the coding sequence with intron(s). For the promoter region, we arbitrary defined a window of 2 kb upstream of the translation start site ATG. Although most TSS are located less than 1 kb of the start codon [50,74], we increased the window to 2 kb, to include *var* promoter regions in their entirety [39], in which canonical G4FS were previously predicted [40]. The intergenic region defines all other regions that are not included in the four categories and may include telomeres and subtelomeres. A second feature list encompassed transcription start sites (TSS), Tn5 hypersensitive sites (THSS), which defined chromatin accessibility sites [49], and *var* genes coordinates. Genomic coordinates of TSS and THSS regions were obtained in TXT format from Adjalley *et al*. [50] and Ruiz *et al*. [49] (clone 10G), respectively. *Var* genes genomic coordinates were extracted from annotation files (excluding those annotated as pseudogenes) and stored as genomic intervals (BED format). TXT and BED files were imported into R to generate the second feature list. For each region, we calculated the total number of G4FS from each G4 list that overlapped with the region coordinates. G4FS intervals that mapped to a promoter region but overlapped with the CDS of the nearest gene were excluded from the analysis. We calculated the density of G4FS in genomic features by dividing the number of G4FS by the feature length and multiplying by 1,000. The three G4 lists were randomly shuffled 1,000 times across the genome and the mean density of randomized G4 lists in genomic features was determined. For each feature category, the ratio of G4FS density over the mean density of random G4FS was calculated and plotted as fold-enrichment. For promoter regions, we computed the distance from a given G4 to the nearest (downstream and upstream) genes and then to retrieve gene information such as geneID, gene product and gene strand using a python script. The list of genes containing at least one G4FS in the promoter region (*i*.*e*., 2 kb upstream region) was curated manually to discard non-coding genes or G4FS within telomeric repeats. Coverage of G4FS within *var* genes was performed with CGAT software [75] using bam2geneprofile tool. All *var* loci, including the 2 kb upstream promoter-containing region, were divided into 1,000 bins and the coverage of G4FS was calculated and normalized within each bin. The heat maps were drawn with R using ggplots (v3.0.1.1). Region intervals from *var* exon 1 encoded domains were retrieved from VarDom server [53]. The 173 recombination breakpoints found to occur during asexual mitotic growth in *P*. *falciparum* [68,69] were obtained from Stanton *et al*. [40]. The mean distance of G4 to the nearest recombination breakpoints was calculated with R and statistical analysis was assessed using the Wilcoxon test. All bioinformatics procedures are described in a R-script provided as a supplementary file (S2 Method).

### G4 oligonucleotides

Oligonucleotides were purchased from Eurogentec (Belgium) and resuspended in bi-distilled water at a concentration of about 500 μM. Concentrations were determined by ultraviolet absorption using molar extinction coefficients provided by the manufacturer. Stock solutions were stored at -20°C.

### Absorbance spectroscopy

Absorption spectra and thermal denaturation profiles (absorbance as a function of temperature) were recorded on an Uvikon XS spectrophotometer (Secoman) coupled to a circulating water bath (Julabo). Measurements were carried out in quartz cells (Hellma) with an optical pathway of 1 cm, according to the following protocol. Each oligonucleotide was dissolved in 10 mM cacodylic acid, adjusted to pH 7.2 with LiOH, supplemented with 100 mM KCl, at a strand concentration of 4 μM. For thermal denaturation profiles, the absorbance was recorded at wavelengths 245, 260, 273, 295 and 335 nm, while cooling the samples from 95°C to 5°C, then heating the samples from 5°C to 95°C at a rate of 0.2°C/min. For all melting curves, cooling and heating profiles were roughly superimposable (no hysteresis was observed). Melting temperatures (*T*_m_) were graphically determined as the intercept between the melting curves and the median lines between low-temperature and high-temperature absorbance linear baselines. Thermal difference spectra (TDS) were obtained by subtracting the absorption spectra at low temperature from the absorption spectra at high temperature, after annealing from high to low temperature at 0.2°C/min [58]. Isothermal difference spectra (IDS) were obtained by subtracting the UV/Vis spectra without and with potassium (100 mM) at 20°C [58]. UV/Vis spectra were recorded on a SAFAS spectrometer.

### Circular dichroism spectroscopy (CD)

Circular dichroism (CD) spectra were recorded on a J-810 spectropolarimeter (Jasco). The oligonucleotide was dissolved at a strand concentration of 3 or 6 μM in 10 mM cacodylic acid, adjusted to pH 7.2 with LiOH and supplemented with 100 mM KCl. CD spectra were recorded at 20°C or 5°C, after annealing from high to low temperature at 0.2°C/min.

### Thioflavin T assay (ThT)

DNA oligonucleotides were prepared at 1 μM in 10 mM cacodylate buffer pH 7.2, supplemented with 100 mM KCl, heated at 95°C for 5 min, then slowly cooled to room temperature in the course of 2 h. The DNA samples were then incubated with 0.5 μM Thioflavin T (3,6-dimethyl-2-(4-dimethylaminophenyl) benzo-thiazolium bromide, 95%; Sigma-Aldrich) at 20°C for 30 min. Fluorescence emission spectra in 450–650 nm wavelength range after excitation at 425 nm were collected on a FluoroMax-4 spectrofluorometer at 20°C. Maximum fluorescence intensity at 490 nm was extracted to analysis the fluorescence enhancement of ThT by the oligonucleotide (FI/F_0_) [59].

### Parasite culture and transfection

*P*. *falciparum* 3D7 strain was maintained in culture in A+ or O+ human RBCs in RPMI-1640 culture medium (Gibco Life Technologies) supplemented with 10% human serum (unless otherwise stated), 0.2 mM hypoxanthine (C.C.Pro GmbH) and 10 μg/mL gentamicin (Sigma) at 5% hematocrit in 5% O_2_/3% CO_2_/92% N_2_ at 37°C. Parasite synchronization was performed by gelatin flotation (Plasmion-based technique) [76] and sorbitol treatment [77]. Synchronized ring-stage parasites (<8 h window) were transfected by electroporation, as described previously [78]. Pyridostatin (PDS) was purchased from Cayman Chemical (USA), resuspended in sterile water at 10 mM (stock solution), and stored at - 20°C, protected from light.

### *In vitro* antimalarial activity of pyridostatin

The effect of PDS on *in vitro P*. *falciparum* growth was measured in microtiter plates according to Desjardins *et al*. [62]. Each well contained 50 μL of complete medium (RPMI 1640 + 10% human serum) in presence or absence of PDS, and 150 μL of *P*. *falciparum*-infected erythrocyte suspension (1.5% final hematocrit and 0.6% parasitemia). The 10 mM stock solution of PDS was diluted in complete medium. After 48 h incubation at 37°C, 0.6 μCi [^3^H]-hypoxanthine were added to each well and the parasites were further incubated for 18 h at 37°C. Cells were lyzed and radioactivity was measured in a liquid scintillation spectrometer (Beckman Coulter). Non-infected erythrocytes were cultured under the same conditions and were used to calculate background radioactivity. Each experiment was performed in triplicate. Results are the mean of three independent experiments. Parasite viability is expressed as 50% parasite growth inhibition (IC_50_).

### Promoter-driven luciferase assay

The plasmids used for luciferase assay were built from the pHLIRH plasmid [79] that encodes for *renilla* gene terminated by the *hrp2* 3’ flanking region [79] and the pHLIRH BSD attP (B) that was derived from pHLIRH and encodes for Firefly luciferase. The Renilla-expressing plasmid was used as internal normalization control. It was constructed by PCR amplification of the promoter region (~1 kb) of the *calmodulin* gene (PF3D7_1434200), from 3D7 genomic DNA, using 5'Cam_ctrl_F and 5'Cam_ctrl_R primers, and cloned into the pHLIRH plasmid using *NcoI*/*SpeI* restriction sites. For the *firefly*-encoding plasmid (pFLuc), the *calmodulin* gene promoter was amplified using 5'Cam_FL_F and 5'Cam_FL_R primers, and cloned into pHLIRH BSD attP (B) using *AscI* and *XmaI* restriction sites. The PF3D7_0800100_pos plasmid was generated by annealing and primer extension of G4C_F and G4C_R primers and their cloning into the pFLuc using *MluI* and *AscI* restriction sites. The PF3D7_0800100_neg and mPF3D7_0800100_neg plasmids were built following the same procedure as PF3D7_0800100_pos but using G4NC_F/G4NC_R primers and mutG4NC_F/mutG4NC_R primers, respectively. PCR amplifications were done with high-fidelity polymerase PfuUltra II Fusion HS DNA polymerase (Agilent Technologies) with elongation temperature of 62°C. Sequence fidelity of the PCR products was verified by capillary sequencing. Cloning reactions were performed using the In-Fusion HD Cloning kit (Clontech) following the manufacturer’s instructions. The list of primers used for all constructs is provided in S7 Table.

Early ring stage parasites (200 μL of infected RBCs at 5–10% parasitemia) were transiently transfected with equal amounts (60 μg) of both Luciferase and Renilla plasmids. The culture medium was changed 24 h after transfection, and at 48 h post-transfection parasites were harvested by saponin-lysis. For the condition with PDS, the G4 ligand was added in the culture medium at a final concentration of 1 μM, immediately following transfection. Samples were resuspended in 50 μL of Passive Lysis Buffer from Dual Luciferase Reporter Assay System (Promega) and disrupted by three cycles of freeze/thaw. Firefly and Renilla luciferase activities were measured using the Dual Luciferase Reporter Assay System according to the manufacturer’s instructions. Mean Firefly luciferase signal in each condition was first normalized to Renilla activity (internal normalization) and then to the control condition without G4 motif in the *calmodulin* gene promoter that was included in all experiments. Results were given as the mean of three independent experiments performed in duplicate and statistical significance was assessed by two-tailed *t* test.

### Parasite synchronisation, RNA extraction and mRNA enrichment

Parasites were thawed from a cryopreserved stock and cultured in O+ erythrocytes (4% hematocrit) under standard conditions with complete medium containing 0.5% AlbuMAX II (Gibco) and 5% human serum for no more than two cycles before the start of the experiment. Highly synchronous cultures were obtained by purification of late schizont stage parasites using Plasmion followed by sorbitol lysis (5 h window). Early ring parasites were diluted at ~6% parasitemia and split into 6 independent cultures. Three biological replicates were kept as untreated control, while 0.6 μM of the G4 ligand PDS was added to the three remaining cultures. Parasites were harvested by saponin-lysis at four time points in the intra-erythrocytic developmental cycle of *P*. *falciparum*: 15 hours post-invasion (hpi; early ring stage), 30 hpi (trophozoite), 40 hpi (schizont) and 58 hpi (early ring stage, 2^nd^ cycle). At the end of the 1^st^ cycle (cycle length ~43 h), the six cultures were diluted 4-fold prior to merozoite reinvasion. Total RNA was extracted using Trizol Reagent (Invitrogen) and contaminating genomic DNA was removed by DNase treatment using TURBO DNA-Free kit (Invitrogen). mRNA was enriched using magnetic oligo-d(T) beads with Dynabeads mRNA Purification kit (Invitrogen).

### Quantitative reverse transcription-PCR (qRT-PCR) on *var* genes

Quantitative PCR was performed on cDNA using specific primers for each *var* gene, as previously described [80] with few modifications from Dzikowski *et al*. [81] (S8 Table). DNase-treated RNA samples were reverse-transcribed into cDNA, using SuperScript III first-strand synthesis SuperMix (Invitrogen), according to manufacturer’s instructions. Alternatively, RNA samples were run without RT enzyme, to check for genomic DNA contamination. Target genes in cDNA samples were quantified using PowerUp SYBR Green Master Mix (Applied Biosystems) and normalized using the housekeeping gene fructose-biphosphate aldolase gene (PF3D7_1444800). The results were expressed as relative copy number.

### Strand specific RNA-Seq library construction and sequencing

mRNA was fragmented and prepared for Illumina library construction as described previously [82]. Briefly, mRNA was fragmented using RNA fragmentation reagents (Ambion) and heating at 70°C for 5 min. Decapping of mRNA was performed by treatment with 10 U RNA 5' Pyrophosphohydrolase (New England BioLabs) for 2 h, at 37°C. Strand specific RNA libraries were prepared using TruSeq Small RNA kit (Illumina) according to the manufacturer’s instructions, with 14 to 16 PCR cycles. Single-end sequencing (50 nt-long) was performed on the Illumina HiSeq 2500 machine.

### RNA-Seq data analysis

Trimmed reads from each FASTQ sample file were mapped to the *P*. *falciparum* 3D7 genome (version 28; [46]) using the BWA-MEM software [83] at default settings. In all samples, we obtained 95% of mapped reads, on average with 24× coverage. To count reads, we used bedtools multicov (v2.26.0). Differential expression analysis was done using DESeq2 (v1.20.0) [84] with the threshold set to adjusted *p*-value <0.01 and >2-fold change of expression. Gene ontology (GO) enrichment analysis of differentially expressed genes was performed using topGO (R package version 2.32.0).

### Validation of RNA-Seq results by quantitative reverse transcription-PCR (qRT-PCR)

DNase-treated RNA samples were reverse-transcribed into cDNA as described above. Target genes in cDNA samples were normalized using the housekeeping gene fructose-biphosphate aldolase gene (PF3D7_1444800) and the 2^-ΔΔCT^ method was used for relative mRNA abundance estimation. The list of primers used for selected genes is provided in S8 Table.

### Data access

The raw sequence data (SRA accession: PRJNA544798) were deposited in the NCBI Sequence Read Archive.

## Supporting information

S1 Method Functions used for G4Hunter analysis in the R script. (R)

S2 Method R script that describes all bioinformatics procedures for G4Hunter search and G4FS distribution analysis. (R)

S1 Table G4 sequences in *P*. *falciparum* genome identified by G4Hunter, using a threshold of 1.2 (G4H1.2), 1.5 (G4H1.5) or 1.75 (G4H1.75) for the G4H score. (XLSX)

S2 Table List of the 108 G4 sequences in *P*. *falciparum* genome predicted by G4Hunter at a threshold of 1.75 that were not found by the G4-seq method. (XLSX)

S3 Table Relative density and mean fold enrichment of G4FS using G4Hunter in different genome features in *P*. *falciparum* with corresponding *p*-values. (XLSX)

S4 Table Genomic coordinates and DNA sequences of G4FS using G4Hunter at threshold 1.2 that are found in gene promoters of the *P*. *falciparum* genome. Promoters with at least 2 G4FS are highlighted in grey. The distance to the nearest CDS was calculated from G4 end position to start codon. The distance to the nearest TSS was calculated from G4 end position to TSS start. *Var* group defines the category of the upstream region (ups) of *var* genes.
(XLSX)

S5 Table List of G4FS that overlap with the coding sequence of *var* genes. For each G4FS, the start and end position are indicated relative to gene feature.
(XLSX)

S6 Table Differentially expressed genes in parasites treated with PDS at different developmental stages (15 h, 30 h, 40 h and 58 h post-invasion). Genes with adjusted *p*-value <0.01 and fold-change of expression >2 compared to non-treated control were considered as significant.
(XLSX)

S7 Table List of primers for cloning of plasmids used in luciferase assay. (XLSX)

S8 Table List of primers for qRT-PCR experiments. (XLSX)

S1 Fig G4 density for each of the 14 chromosomes in the *P*. *falciparum* 3D7 genome calculated using different thresholds (G4H1.2, G4H1.5, G4H1.75). The dotted lines represent the G4 density for the whole genome. Significant *p*-values (p<0.05) are indicated above the chromosomes.
(TIF)

S2 Fig Density and coverage of *var* genes (A) and transcript (B) on the 14 chromosomes of *P. falciparum* genome. Grey bars represent feature density and coverage on both strands. Pink and blue bars represent feature density and coverage on coding and non-coding strands, respectively.
(TIF)

S3 Fig Distribution profile of G4 motifs (G4FS) within 1 kb of transcription start site (TSS). The red and blue lines correspond to G4FS found in the coding and non-coding strands, respectively.
(TIF)

S4 Fig G4FS frequency in *var* negative and positive strands at threshold 1.2. The metagene plots illustrate the G4FS frequency in the negative and the positive strands of the *var* gene repertoire. The 2-kb promoter region is delimited by the dotted red line.
(TIF)

S5 Fig G4 density for the 14 chromosomes of *P. adleri* (A), *P. billcollinsi* (B), *P. blacklocki* (C), *P. praefalciparum* (D) and *P. reichenowi* (E) at thresholds 1.2. The dotted lines represent the G4 density for the whole genome.
(TIF)

S6 Fig Fluorescence enhancement of ThT in the presence of selected G4 oligonucleotides. The fluorescence enhancement was calculated by dividing the fluorescence intensity of ThT in presence of oligonucleotides at 490 nm (FI) by the fluorescence intensity of ThT alone (FI_0_). The c-Myc and dT30 sequences were used as positive and negative controls, respectively. The sequences of oligonucleotides are shown in Table 3. Error bars correspond to the standard deviation.
(TIF)

S7 Fig Thermal denaturation curves of three G4FS in *var* promoters. The measurements were carried out in 100 mM KCl at 6 μM strand concentration.
(TIF)

S8 Fig *Var* gene expression profiles in untreated (filled bars) and PDS-treated (dashed bars) *P. falciparum* parasites at ring stage (2^n^d cycle). Histogram colours from yellow to grey indicate different categories of 5’ upstream flanking region (ups), based on sequence and chromosomal location. Results are expressed as relative copy number and the fructose-biphosphate aldolase gene (PF3D7_1444800) was used as internal control. The results are the mean of three biological replicates performed in triplicate. G4-containing promoters are highlighted in bold.
(TIF)
