# Supplementary figures and images for: Genome wide distribution of G-quadruplexes and their impact on gene expression in malaria parasites
Source: PLoS Genet. 2020 Jul 6;16(7):e1008917. doi: 10.1371/journal.pgen.1008917 (PMC7365481; doi:10.1371/journal.pgen.1008917)

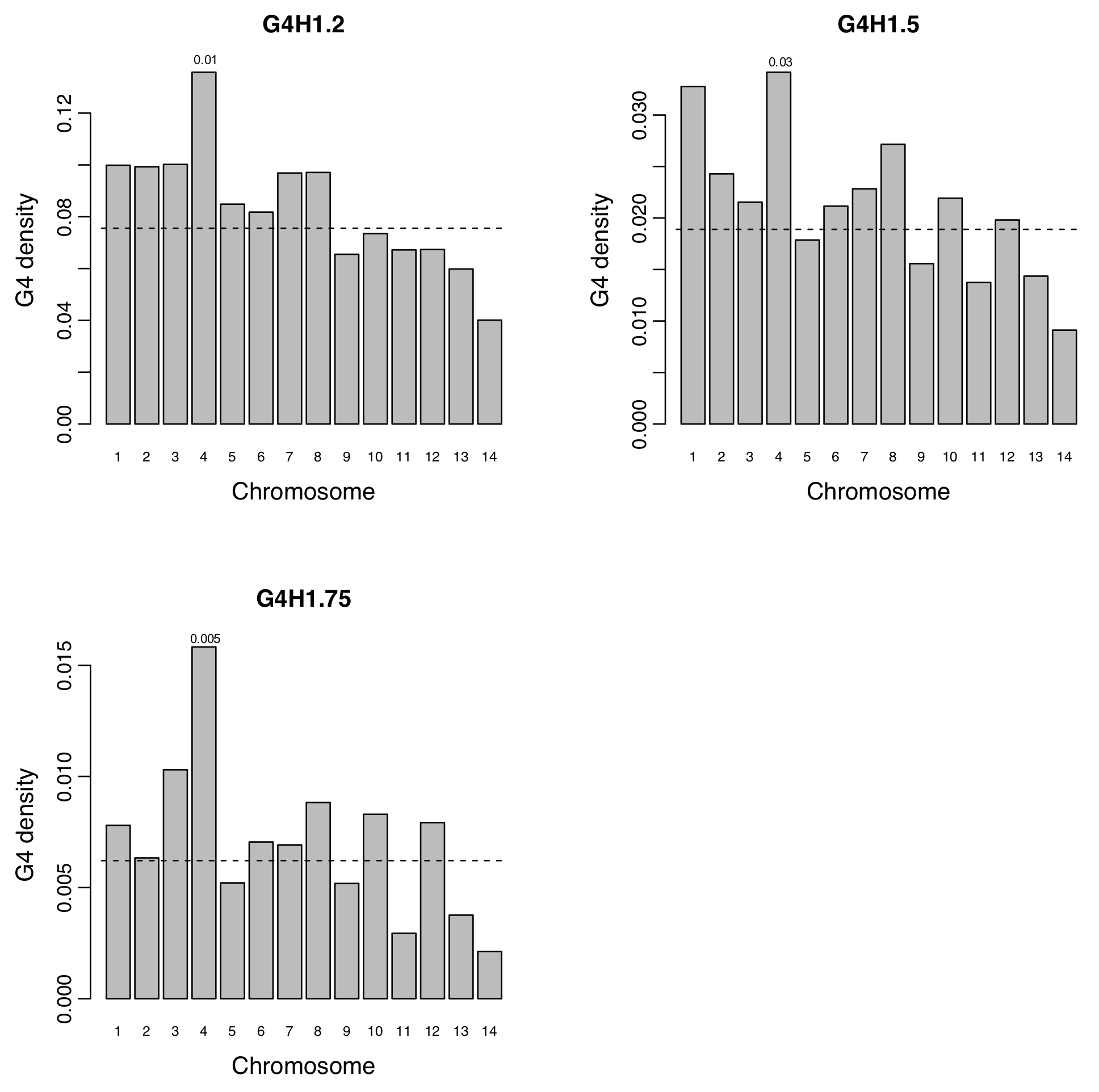

Supplement: S1 Fig — The dotted lines represent the G4 density for the whole genome. Significant p-values (p<0.05) are indicated above the chromosomes. (TIF) [file pgen.1008917.s011.tif]

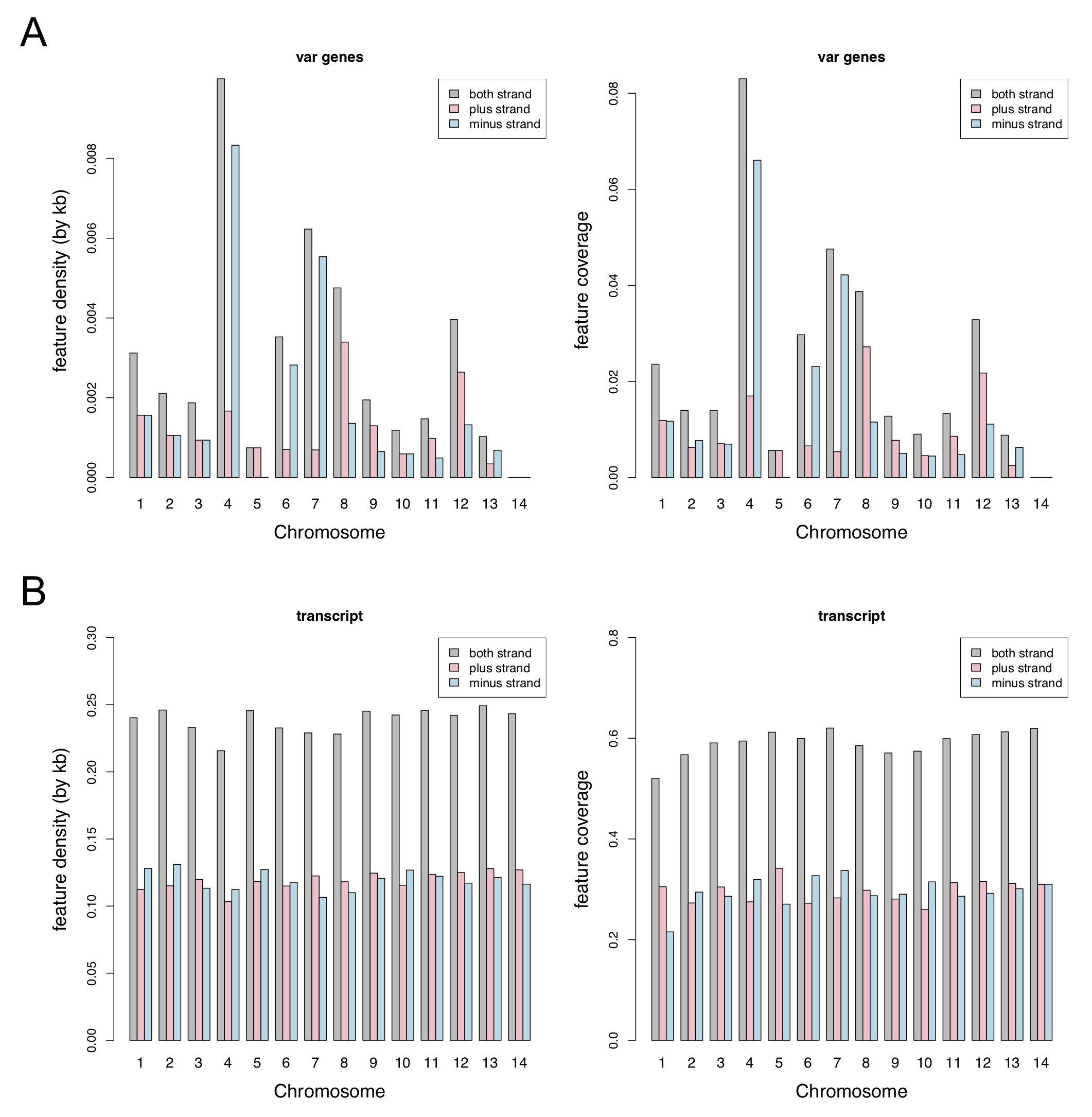

Supplement: S2 Fig — Density and coverage of var genes (A) and transcript (B) on the 14 chromosomes of P. falciparum genome. Grey bars represent feature density and coverage on both strands. Pink and blue bars represent feature density and coverage on coding and non-coding strands, respectively. (TIF) [file pgen.1008917.s012.tif]

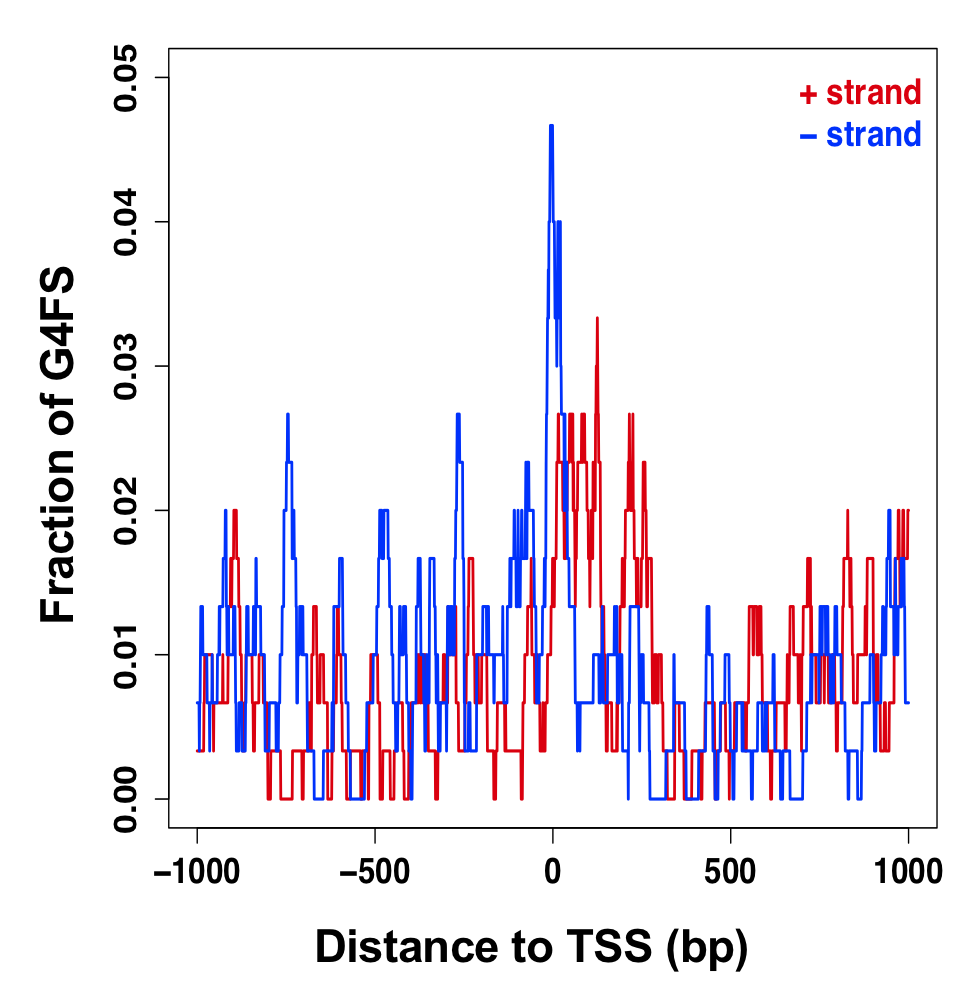

Supplement: S3 Fig — The red and blue lines correspond to G4FS found in the coding and non-coding strands, respectively. (TIF) [file pgen.1008917.s013.tif]

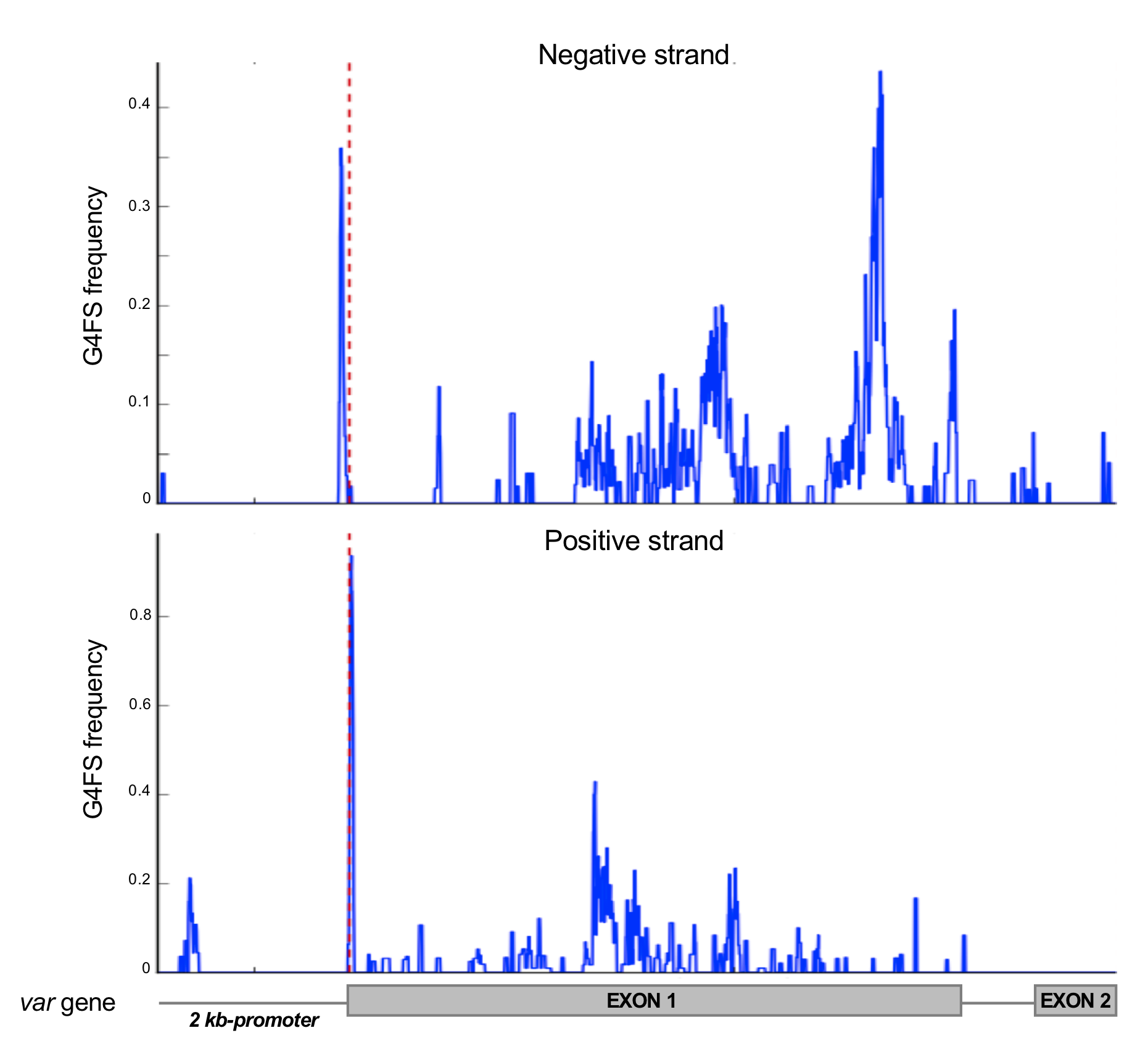

Supplement: S4 Fig — The metagene plots illustrate the G4FS frequency in the negative and the positive strands of the var gene repertoire. The 2-kb promoter region is delimited by the dotted red line. (TIF) [file pgen.1008917.s014.tif]

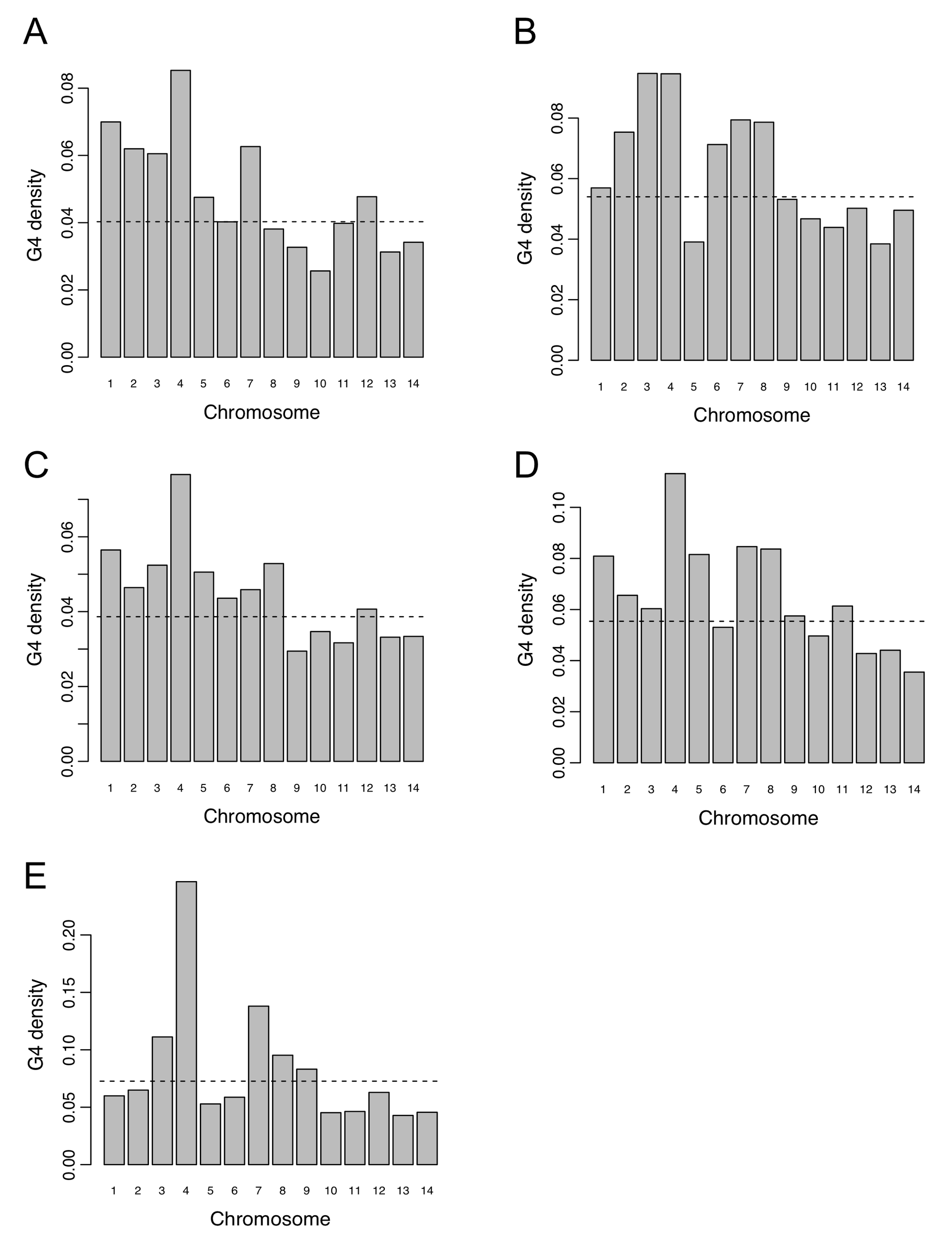

Supplement: S5 Fig — G4 density for the 14 chromosomes of P. adleri (A), P. billcollinsi (B), P. blacklocki (C), P. praefalciparum (D) and P. reichenowi (E) at thresholds 1.2. The dotted lines represent the G4 density for the whole genome. (TIF) [file pgen.1008917.s015.tif]

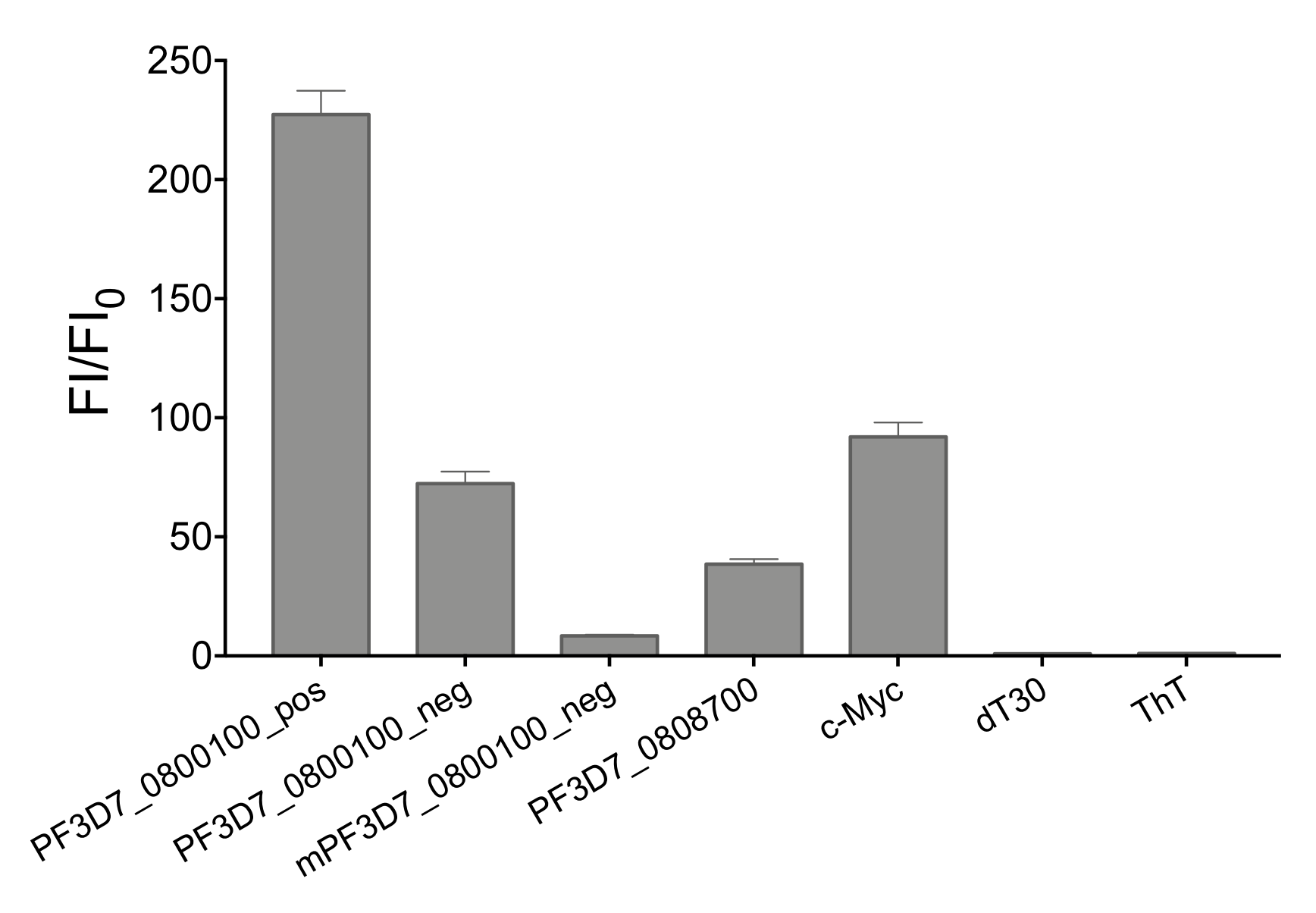

Supplement: S6 Fig — The fluorescence enhancement was calculated by dividing the fluorescence intensity of ThT in presence of oligonucleotides at 490 nm (FI) by the fluorescence intensity of ThT alone (FI0). The c-Myc and dT30 sequences were used as positive and negative controls, respectively. The sequences of oligonucleotides are shown in Table 3. Error bars correspond to the standard deviation. (TIF) [file pgen.1008917.s016.tif]

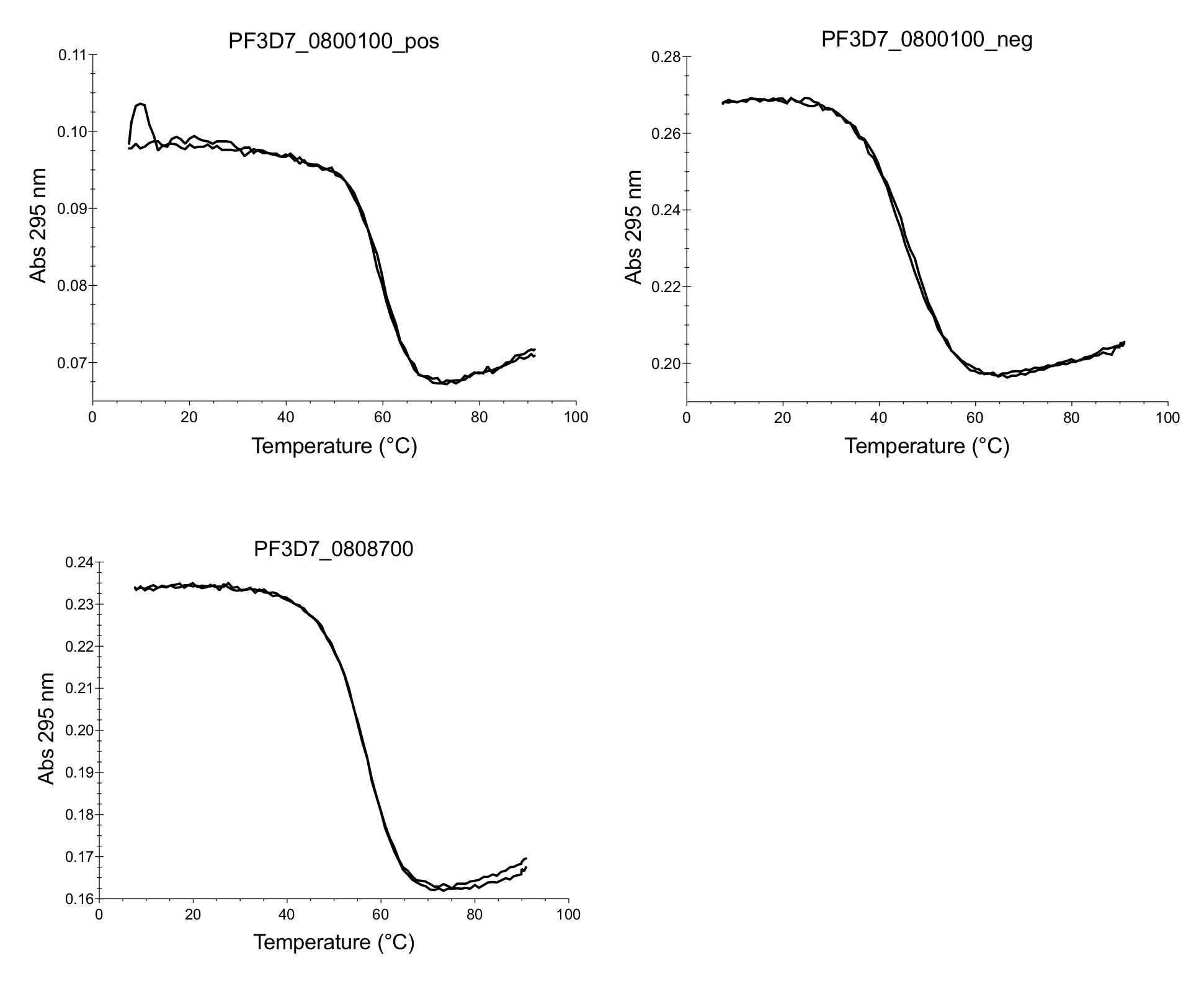

Supplement: S7 Fig — The measurements were carried out in 100 mM KCl at 6 μM strand concentration. (TIF) [file pgen.1008917.s017.tif]

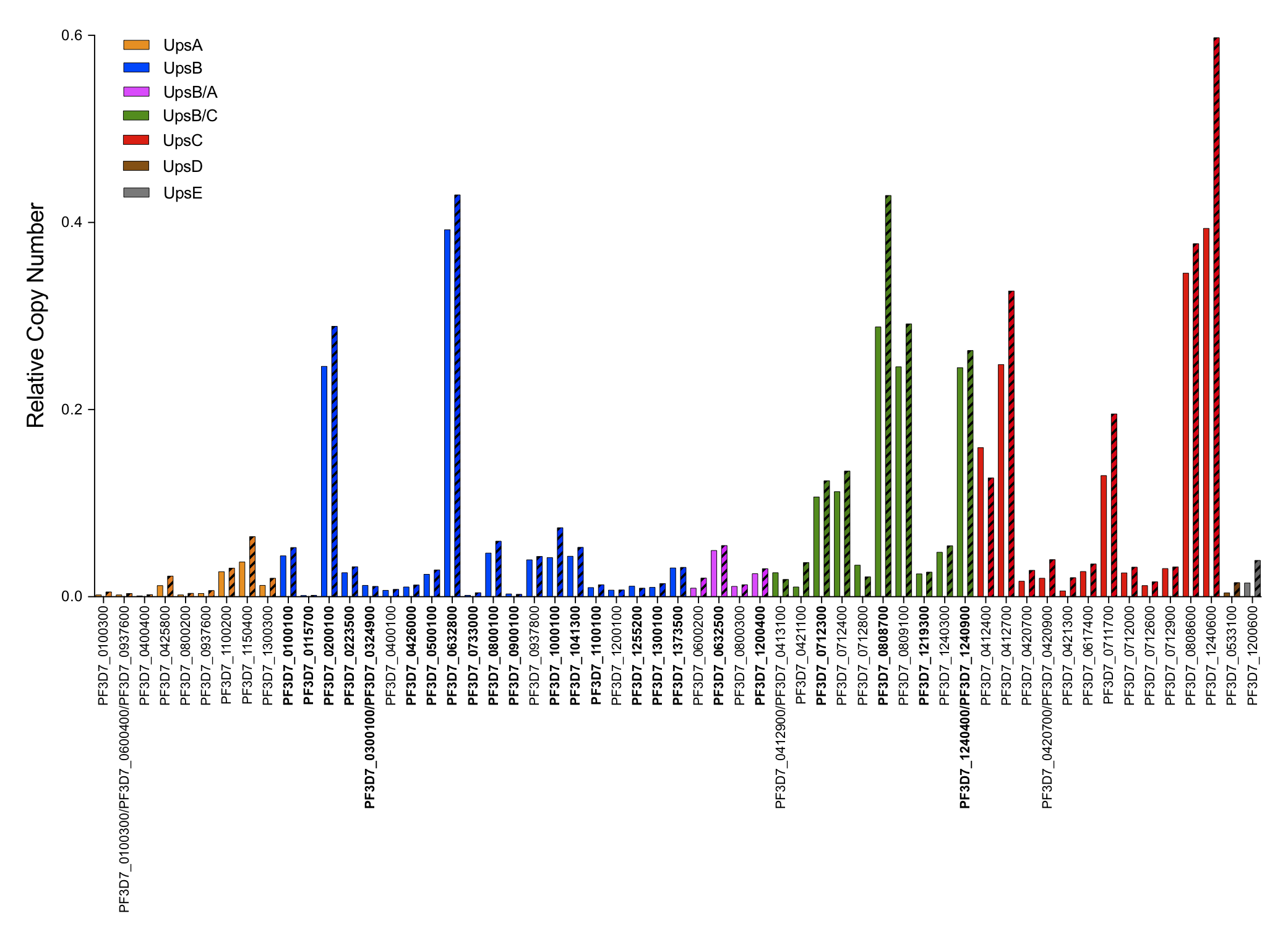

Supplement: S8 Fig — Var gene expression profiles in untreated (filled bars) and PDS-treated (dashed bars) P. falciparum parasites at ring stage (2nd cycle). Histogram colours from yellow to grey indicate different categories of 5’ upstream flanking region (ups), based on sequence and chromosomal location. Results are expressed as relative copy number and the fructose-biphosphate aldolase gene (PF3D7_1444800) was used as internal control. The results are the mean of three biological replicates performed in triplicate. G4-containing promoters are highlighted in bold. (TIF) [file pgen.1008917.s018.tif]
